# Nano-oncology revisited: Insights on precise therapeutic advances and challenges in tumor

**DOI:** 10.1016/j.fmre.2025.03.024

**Published:** 2025-05-14

**Authors:** Lesheng Teng, Ye Bi, Xiaofang Xing, Gang Yao

**Affiliations:** a School of Life Sciences, Jilin University, Changchun 130012, China; b Department of Health Sciences, National Natural Science Foundation of China, Beijing 100085, China; c Public Experimental Center, Changchun University of Chinese Medicine, Changchun 130117, China; d Department of Gastrointestinal Cancer Center, Peking University Cancer Hospital & Institute, Beijing 100142, China

**Keywords:** Nano-oncology, Personalized perspective, Nanomedicine, Multi-stage targeting strategies, Artificial intelligence

## Abstract

The integration of nanotechnology with cancer therapy has revolutionized traditional diagnostic and treatment methods, giving rise to the new interdisciplinary field of nano-oncology. With advancements in scientific research and ongoing technological innovations, nano-oncology is expected to play an increasingly critical role in future cancer therapies. However, the clinical translation of nanomedicines still faces several challenges, including the interaction of nanoparticles with biological systems, effective accumulation at tumor sites, and the precise fabrication of therapeutic agents. This review focuses on the challenges and future directions in nano-oncology research, with a specific emphasis on multi-stage targeting strategies and the application of artificial intelligence. The aim is to provide insights into the development of individualized nanomedicine approaches and contribute to advancing the field of nano-oncology.

## 1 Introduction

Nano-oncology, an emerging interdisciplinary field that integrates nanotechnology, tumor biology, drug delivery, molecular imaging, and biomaterials, holds tremendous potential for revolutionizing novel diagnostic and therapeutic strategies by combining cancer biology with nanotechnology. The origins of nano-oncology date back to the late 20th century, when scientists began to explore nanoscale materials and their applications in the biomedical field [1]. From the late 1990s to the early 2000s [2], research efforts were focused on applying nanotechnology to cancer treatment, primarily investigating the fundamental properties of nanomaterials as potential drug carriers. Significant advances in nano-oncology occurred between 2000 and 2010, including the development of intelligent nanodrug delivery systems, multidrug nanomedicines, and targeted nano-therapies [3]. These innovations facilitated precise tumor-targeting treatments, yielding promising results in preclinical animal models. Table S1 summarizes the currently widely used nano-dosage forms and their applications in cancer treatment and diagnosis. Since 2010, continued advancements in nanotechnology have facilitated the clinical translation of nano-oncology [4]. Several nanomedicine formulations have gained regulatory approval for the treatment of specific cancers [5], and nanomedical imaging technologies have shown great promise in early tumor diagnosis and monitoring of treatment efficacy.

The rapid development of nano-oncology over the past three decades has not only driven innovations in cancer treatment but also provided new insights into early cancer detection and prognosis assessment. With advancements in scientific research and ongoing technological innovations, nano-oncology is expected to play an increasingly pivotal role in future cancer therapies. Current key research directions in this field include nanodrug delivery, intelligent nano-diagnostic and therapeutic systems, tumor nano-immunotherapy, and biomimetic nanodrug delivery systems.

However, existing research reveals persistent challenges and issues. In some cases, the pursuit of novel nanomaterials has placed excessive emphasis on innovation while overlooking the practical clinical translation of these materials. Additionally, limited expertise in integrating interdisciplinary technologies and methods restricts the effectiveness of cross-disciplinary collaboration. Insufficient attention to niche research areas may further hinder potential breakthroughs.

This review highlights the significance and clinical challenges of nano-oncology in cancer treatment. Moreover, it provides a comprehensive overview of future research priorities in nano-oncology, with a particular focus on the dynamic integration of multi-stage tumor-targeting strategies, the potential role of artificial intelligence (AI) in optimizing nanoparticle delivery to solid tumors, and the prospects of nanomedicine for personalized treatment. Through these comprehensive analyses, this review aims to serve as a valuable reference for researchers and propose actionable guidance for future research in tumor nanoscience.

## 2 Research status and trends of nano-oncology

### 2.1 Selective targeting-based nanomedicine: tumor tissues, tumor microenvironment (TME), tumor cells, and organelles

**Fig. 1:**
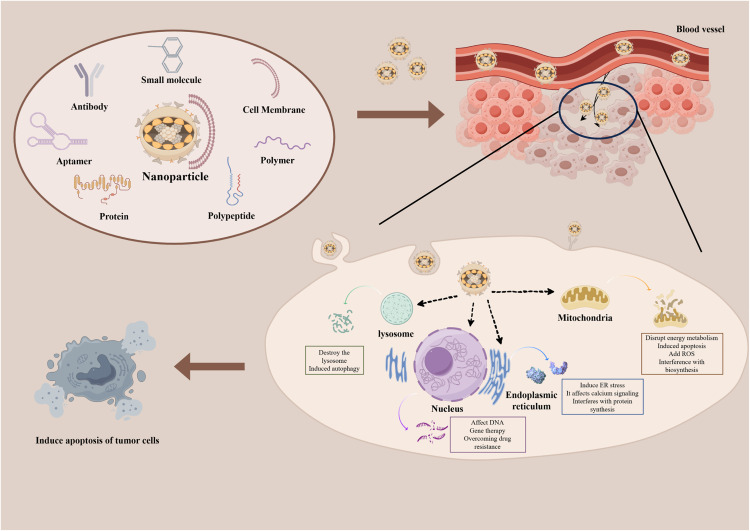
**Selective targeted drug delivery strategies focus on tumor tissues, TME, tumor cells, and organelles**.

With the deepening of research in cancer and nanomedicine, the research focus has shifted toward the efficient and precise delivery of nanomedicine to tumor sites. This strategy aims to minimize nonspecific drug toxicity by targeting tumor tissue, the TME, specific cells, and organelles while maximizing therapeutic efficacy. Fig. 1 illustrates established selective targeted drug delivery strategies.

Enhancing the permeability and retention (EPR) effect, a long-established strategy, can increase the accumulation of nanomaterials in tumors. However, the highly heterogeneous nature of the EPR effect poses a significant challenge to the clinical application of nanomedicine. Despite the development of some auxiliary methods, certain tumors with non-leaky vasculature remain resistant to the EPR effect. Consequently, several alternative strategies have been explored, including tumor vascular targeting, live cell-mediated targeting, iRGD-mediated tumor targeting, and local/regional delivery [6]. To further enhance efficacy, nanomedicine must achieve higher precision and specificity in targeting tumor cells. Through rational design, ligands such as antibodies, antibody fragments, nucleic acid aptamers, peptides, carbohydrates, and small molecules can be engineered to specifically bind to receptors on cancer cells. These ligands promote nanocarrier uptake, directly induce apoptosis in cancer cells, and minimize off-target toxicity to normal cells.

To ensure internalization by tumor cells, the impact of the TME—an inherent physiological barrier—cannot be overlooked in clinical therapy. This complex microenvironment consists of diverse cell types, vascular structures, extracellular matrix components, and biochemical factors, forming a dynamic and heterogeneous ecosystem. The distribution and permeability of nanomaterials are influenced by the acidic environment, hypoxia, high interstitial pressure and other factors in TME, which require the nanoparticles to exhibit sufficient stability and adaptability [7]. Fortunately, these challenges also present therapeutic opportunities, such as designing pH-responsive nanoparticles that leverage the acidic TME to control drug release. Furthermore, components such as collagen fibers and glycosaminoglycans within the tumor extracellular matrix create a physical barrier that impedes nanoparticle penetration [8]. To enhance nanoparticle penetration, researchers employ strategies such as applying specific charges or targeting ligands, adjusting the physical properties of nanoparticles, and degrading collagen fibers [9]. However, the collagen composition is complex, and current research remains limited. A deeper understanding of its fundamental mechanisms is essential for the rational design of more effective nanocarriers.

It should not be overlooked that immune cells and various other cell types can interact with nanoparticles through processes such as endocytosis or degradation, potentially activating the immune system and influencing nanoparticle retention. These interactions may lead to nanoparticle recognition and clearance by the body, thus limiting therapeutic efficacy [10]. Therefore, reprogramming the TME by targeting key cellular components, such as immunosuppressive cells, has emerged as a logical and promising therapeutic approach [11]. Biochemical factors and signaling pathways in the TME also influence the distribution and therapeutic effects of nanomaterials [12], necessitating a deeper understanding of the underlying mechanisms to design more effective and targeted nanoparticles. Traditional methods for studying the TME and tumors remain limited, driving the development of novel approaches. Microfluidic human tumor chips and three-dimensional (3D) *in vitro* models can simulate key aspects of the human TME and vasculature, thereby enhancing our understanding of nanoparticle tumor extravasation in patients [13]. By integrating these strategies, researchers aim to gain deeper insights into the TME, ultimately leading to the development of more effective nanotherapeutic strategies and offering new hope to cancer patients.

After penetrating the complex barrier of the TME, nanomedicine can selectively accumulate in tumor tissue but may not effectively enter tumor cells. To achieve precise treatment, nanocarriers require specific modification through engineered targeting mechanisms. Commonly used targeting ligands include antibodies, aptamers, peptides, carbohydrates, and small molecules, which bind to specific antigens or receptors on the tumor cell surface. Among these, antibodies and peptides are the most widely used ligands for tumor targeting. Prominent targets include epidermal growth factor receptor (EGFR), human epidermal growth factor receptor-2 (HER2), and prostate-specific membrane antigen (PSMA). Antibody-based therapies targeting these receptors have shown remarkable efficacy [14]. Aptamers, single-stranded DNA or RNA molecules with high specificity and affinity, function similarly to traditional antibodies and can target markers such as PSMA and epithelial cell adhesion molecule [15]. They offer advantages such as small size, high stability, easy modification, minimal batch-to-batch variation, and low immunogenicity. Carbohydrates, owing to their favorable biocompatibility and specific recognition of cell surface proteins, have been widely employed as targeting ligands in nanoparticle delivery to tumor cells. Carbohydrates like hyaluronic acid, galactose, and their derivatives can promote efficient delivery by specifically binding to receptors such as lectins [16]. In recent years, small molecules like folic acid and biotin have been employed as ligands for the targeted delivery of nanomedicine to tumors. Additionally, nanoparticles coated with natural or biomimetic membranes can be endowed with homologous or allogeneic adhesion properties derived from original cells, constituting natural targeting strategies based on biological principles. This approach not only enhances nanoparticle targeting but also provides a novel paradigm for the design of nanoscale drug delivery systems.

Following internalization into target cells, nanomaterials must be precisely delivered to their site of action. The primary goal is to trigger cell death signal pathways through precise accumulation in key organelles, thereby minimizing off-target toxicity to normal cells and reducing the required drug dosage [17]. In this approach, organelles such as the nucleus, mitochondria, lysosomes, and endoplasmic reticulum (ER) are critical targets.

The cell nucleus plays a central role in cancer initiation and development, with genetic mutations and chromatin remodeling serving as crucial factors. The cell cycle, apoptosis, and aging processes—regulated by the nucleus—are often inhibited in cancer, allowing cancer cells to survive and proliferate. Additionally, the nucleus contributes to drug resistance, enabling cancer cells to resist chemotherapy agents. Thus, it represents a key target for cancer gene therapy and research. However, the hydrodynamic diameter of most nanoparticles exceeds the size limit of nuclear pore complexes, restricting their ability to target the nucleus [18]. To overcome this obstacle, current strategies primarily involve modifying the nanoparticle surface with small molecules, aptamers, or other agents that promote nuclear pore dilation, thereby facilitating nanoparticle entry into the nucleus [19]. Despite these efforts, the mechanisms underlying nuclear entry remain incompletely understood. Moreover, the complex substructures within the nucleus, such as chromatin and nucleolus, further complicate precise nanoparticle localization.

Mitochondria, often referred to as the “suicide weapon stores” of eukaryotic cells, serve as convergence points for various lethal signal transduction cascades, making them a promising target of significant interest. Mitochondrial targeting signals/sequences, mitochondrial penetrating peptides, triphenylphosphine, DQAsomes, and MITO-Porter all play significant roles in specifically targeting tumor cell mitochondria. However, the DNA repair mechanisms within mitochondria enable them to evade the consequences of nanoparticle-induced mitochondrial DNA damage [20]. Therefore, it is critical to develop strategies that simultaneously disrupt multiple mitochondrial transcription and translation components for enhancing efficacy.

Lysosomes play a vital role in regulating autophagy and cell death pathways, thereby affecting cancer cell survival and tumor progression by releasing signaling molecules and maintaining an acidic environment. This underscores the importance of lysosomes as a therapeutic target in cancer. Targeting lysosomes in cancer cells inhibits tumor growth, invasion, and metastasis by disrupting degradation functions, inducing cell death, increasing intracellular stress, inhibiting TME modification, and enhancing drug sensitivity. Ultimately, this approach aims to improve cancer treatment outcomes and patient survival. Currently, nanoparticles can target endosomal and lysosomal compartments through endocytic pathways, lysosomal sorting peptides, morpholine, and other strategies to exert anti-cancer effects [21]. However, it is important to recognize that extracellular materials are readily degraded in lysosomes once internalized by tumor cells. Thus, achieving lysosomal escape is crucial for maximizing the therapeutic potential of nanotechnology in cancer therapy.

The endoplasmic reticulum, in addition to its essential role in protein synthesis and modification, also plays a crucial role in regulating cellular stress responses and calcium signaling. Dysregulation in ER morphology and function in cancer cells contributes to tumor growth, invasion, metastasis, and altered drug sensitivity. Targeting the ER in cancer cells can promote cell death through multiple mechanisms, including disruption of protein synthesis, induction of apoptosis, modulation of tumor metabolism, enhancement of drug sensitivity, damage to cellular structure, and regulation of signal transduction pathways, ultimately improving the efficacy of cancer treatments [22].

However, organelle targeting still faces numerous challenges. The crowded and complex cellular environment limits nanoparticle delivery. The organelle membrane barrier hinders nanoparticle penetration. In addition, the complexity and interaction of organelles make it difficult to achieve the desired therapeutic outcomes [23]. The complex endocytosis mechanism of nanoparticles and the occurrence of non-specific endocytosis reduce drug targeting efficiency. To overcome these challenges, further research is needed to investigate the endocytosis mechanisms of organelles, design nanoparticles with high permeability, design ligands that specifically bind to organelles, and deepen the study of cell signaling and molecular pathways to identify more effective subcellular targets. Additionally, the therapeutic safety of organelle-targeting cancer therapies requires further evaluation.

Despite the abundance of nanoparticle-based targeting strategies in cancer therapy, clinical effects have fallen short of expectations, typically serving only to reduce drug side effects. The complex physiological environment severely impacts the therapeutic efficacy of nanomedicine, highlighting the urgent need for more scientifically sound and rationally designed strategies. Future efforts should prioritize developing therapies capable of precisely identifying and targeting tumor cells, enabling personalized and efficient cancer treatment.

### 2.2 Smart nano-systems

The precise delivery of nanoparticles in cancer therapy faces numerous challenges. Traditional nanoparticles exhibit limited adaptability to environmental changes, and their targeting and release control functions are constrained, resulting in poor *in vivo* distribution accuracy and limiting multi-functional integration. These limitations hinder the clinical translation of nanoparticles. In response to these limitations, smart nanoparticles, which respond to specific environmental stimuli to activate therapeutic functions or release drugs, are gaining significant attention due to their numerous advantages. Smart nanoparticles are designed to supersede traditional nanoparticles, with their core feature being stimulus-responsive drug delivery mechanisms. These mechanisms can achieve precise drug delivery upon exposure to specific biological signals, thereby enhancing therapeutic efficacy and minimizing off-target effects. This mechanism effectively addresses the issue of premature or incomplete drug release in non-target areas, introducing a novel and intelligent model for drug delivery systems [24].

In smart nanoparticle research, the development of functionalized modules and materials is crucial for diverse delivery strategies. Smart nanoparticle carriers encompass various forms, including lipid-based carriers, polymer nanoparticles, micelles, self-assembled chemical drugs, nucleotide-based carriers, self-assembling peptide systems, and cell-derived or biomimetic delivery systems. To optimize therapeutic outcomes, researchers must select the most suitable nanoparticle carrier based on disease type and the characteristics of the drug being delivered.

The effectiveness of smart nanoparticles depends on both endogenous stimuli (*e.g.*, pH, enzymes, and redox reactions) and exogenous stimuli (*e.g.*, temperature, light, ultrasound, electric fields, and magnetic fields), enabling spatial, temporal, or dose-controlled drug delivery. Biomimetic carriers, a key focus in smart nanoparticle research, introduce innovations in drug delivery and disease treatment through their unique biomimetic properties. By mimicking natural structures, these carriers effectively mitigate traditional nanoparticle challenges, such as rapid clearance from circulation, immune system recognition, and insufficient target accumulation [25].

The diverse design of smart nanoparticles not only optimizes existing therapeutic drugs but also promotes the development of combination therapies and multi-responsive platforms [26]. By creating comprehensive nanomedicine libraries and employing systems that screen multiple parameters through advanced techniques like microfluidics, researchers have developed the next generation of smart nanoparticles [27]. Additionally, the integration of computer-aided design and AI enhances the potential and complexity of smart nanoparticles. Moreover, smart nanoparticles are often multifunctional, integrating both imaging and therapeutic effects to enable theranostics. This capability allows real-time monitoring of treatment processes and effectiveness, optimizing cancer treatment strategies and enhancing treatment precision and safety.

However, the *in vivo* toxicity of nanomedicines remains a significant barrier to the successful application of smart nanoparticles. Despite extensive *in vitro* and animal toxicity studies, the relative scarcity of human studies limits their broader application [28]. Challenges persist in designing biologically functional and controllable components, such as predicting *in vivo* stimulus behavior, standardizing evaluation criteria, and facilitating clinical translation and industrial production.

### 2.3 Multidrug nanomedicine

In cancer treatment, single-drug therapies face the dual challenges of drug resistance and limited mechanisms of action, restricting their efficacy against complex cancer dynamics. The heterogeneity and dynamics of tumor cells make it difficult to counteract the development of multiple resistance mechanisms against single-drug effects [29]. As a result, single-drug treatments often exhibit limited efficacy due to their inability to address tumor cell diversity and adaptability. To overcome these limitations, multidrug nanocarriers have emerged as a promising strategy. By delivering multiple drugs simultaneously, this approach targets tumor cells through diverse mechanisms, effectively mitigating the limitations of single-drug therapies.

However, researchers face challenges in determining whether the proportion of free drugs influences drug loading in nanocarriers and whether multidrug nanocarriers outperform mixtures of single-drug nanocarriers at equivalent doses. Recently, Detappe et al. [30] developed a bottlebrush prodrug (BPD) platform for delivering three drugs to tumors. These three BPDs can be readily formulated in proportion, generating random mixtures of mono-, di-, and tri-drug-loaded nanoparticles that yield synergistic therapeutic effects in a mouse model of multiple myeloma. Unlike traditional multi-drug nanoparticles, this design enables adjustable drug ratios within a single nanocarrier, paving the way for more precise nanoparticle designs in the future.

Current challenges for multidrug nanoparticles include optimizing drug loading, release, and distribution; characterizing and evaluating nanocarriers and their interactions with biological systems; controlling the release rates of multiple drugs; and regulating and standardizing nanomedicine products [31]. Artificial intelligence significantly impacts multidrug nanomedicine, providing crucial assistance in drug selection. With advances in nanotechnology, biotechnology, and pharmaceutical sciences, multifunctional nanomedicine is poised to revolutionize the diagnosis and treatment of various diseases in the near future.

## 3 Obstacles in a forward vision for nano-oncology

**Fig. 2:**
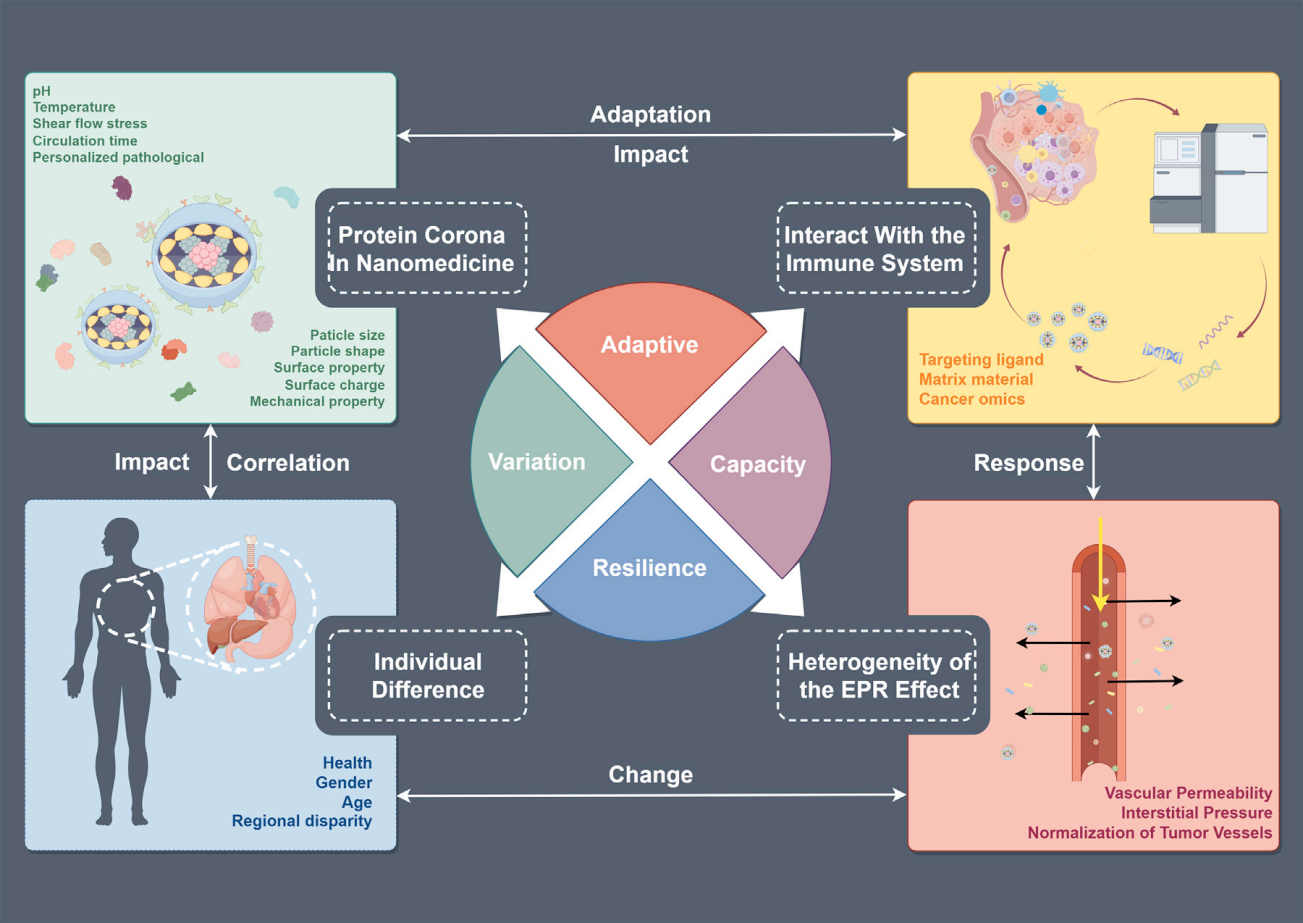
**Obstacles in a forward vision for nano-oncology include protein corona, interaction with the immune system, individualized differences, and the EPR effects *in vivo***.

As previously discussed, nano-oncology represents the revolutionary application of nanobiotechnology in the diagnosis, treatment, and prevention of cancer. It holds the potential to significantly improve the efficacy and safety of cancer therapies while enabling novel diagnostic and therapeutic modalities. Nevertheless, nano-oncology confronts significant obstacles stemming from the complexity and heterogeneity of the TME [32], as well as the complex interactions between nanoparticles and biological systems. The common obstacles in nano-oncology, outlined in Fig 2, are described in detail below.

### 3.1 How many nanoparticles could accumulate in a tumor, on-target or off-target?

Nanoparticles serve as effective vectors for delivering cytotoxic drugs or imaging agents to tumor sites. These nanoparticles can carry multiple therapeutic or imaging molecules, preserving their biological activity and increasing their solubility in biological fluids. A range of factors, including nanoparticle size, shape, surface characteristics, and targeting ligands, as well as the extracellular matrix, stromal cells, interstitial pressure, and vascular permeability within the TME, affect the extent of nanoparticle accumulation in tumors [33]. One study reported that the average accumulation of nanoparticles in solid tumors is approximately 0.7% of the injected dose per gram of tumor tissue, which is considerably lower than the desired therapeutic dose of 10% [34].

To enhance nanoparticle accumulation in tumors, various strategies have been proposed, including optimizing nanoparticle characteristics, improving tumor vasculature, disrupting the tumor matrix, and modulating tumor-stroma interactions [35]. However, the variability of tumor types, the heterogeneity of the EPR effect [33], and the potential adverse effects of the TME disruption pose additional challenges to these strategies.

Thus, nanoparticle accumulation in tumors remains a challenge due to its inherent unpredictability, as it depends on both the specific characteristics of the tumor and the nanoparticle delivery system. Further research is necessary to optimize nanoparticle design and delivery strategies, as well as to better understand the complex interplay between nanoparticles and the TME.

#### 3.1.1 The environmental science of protein corona in nanomedicine

Protein coronas are defined as layers of protein molecules adsorbed onto the surface of nanoparticles in biological environments. These coatings can alter nanoparticle behavior in various parts of the body, influencing characteristics such as toxicity, clearance rate, biodegradability, aggregation, size, shape, charge, stability, and therapeutic efficacy [36]. Moreover, protein coronas influence nanoparticle interactions with the immune system, tissues, cells, organs, and their surrounding environment.

Several factors influence the composition of the protein corona, including the physicochemical properties of the nanoparticles, the type and quantity of proteins, the duration and conditions of exposure, and the variability of the biological system. For instance, hydrophobic nanoparticles surfaces tend to adsorb hydrophilic proteins more efficiently—by a factor of 2.1—due to hydrophobic interactions [37]. The surface chemistry of nanoparticles significantly influences the formation and dynamics of the protein corona. This protein corona exhibits significant differences between *in vitro* and *in vivo* conditions owing to its highly dynamic and complex nature. *In vivo*, protein coronas evolve dynamically, particularly with regard to the rate of protein exchange between nanoparticles and the surrounding fluid. This dynamic evolution can provide insights into nanoparticle interactions with specific receptors and their circulation time in the bloodstream [38]. Most adsorbed proteins are characterized by low molecular weight and negative charge due to the curvature of the nanoparticle surface and electrostatic interactions. Consequently, understanding and controlling the formation and evolution of the protein corona is critical for advancements in nanomedicine and nanotechnology.

Several strategies have been developed to modify protein coronas, offering opportunities for nanoparticle engineering and design. These strategies encompass surface modification, application of active or stealth coatings, exploitation of TME features, and adoption of pre- or post-coating methods [36]. Nanoparticles with coronas enrich in opsonins, such as complement factors, fibrinogen, or IgG, are rapidly cleared from the body through macrophage recognition and subsequent phagocytosis [39]. In contrast, nanoparticles with coronas rich in dysopsonins, such as albumin or apolipoproteins, tend to exhibit prolonged circulation times [40]. Therefore, nanoparticle design should prioritize enhancing dysopsonins adsorption while minimizing overall protein adsorption to improve tissue compatibility. The dynamic formation of the protein corona in biological fluids can be mathematically modeled and extensively investigated across various parameters [38].

Some studies suggest that protein coronas can enhance nanoparticle stability, biodegradability, and therapeutic efficacy, whereas others emphasize their potential to cause mistargeting, unexpected toxicity, and reduced therapeutic efficacy. Research on protein coronas encounters several challenges, including the complexity and variability of biological systems, the absence of standardized techniques and protocols, and the difficulty in predicting and controlling corona formation and evolution. Additionally, ethical and regulatory considerations surrounding nanoparticle use in living organisms and the environment add further complexity. Therefore, understanding the role of protein corona is crucial for the development and application of nanomedicine and nanotechnology. Further research is imperative to enhance our understanding of the functions and mechanisms of protein coronas, and to develop novel tools and methods for their manipulation and application in environmental science and nanomedicine.

#### 3.1.2 The fate of systemically administered nanomedicine: the hide-and-seek interaction between nanoparticles and the immune system

The immune system, a crucial mediator of nanoparticle interactions, comprises a highly coordinated network of cells capable of recognizing foreign substances. Components of the immune system can either act as barriers to nanoparticle delivery or serve as targets, eliciting signaling cues that enhance systemic therapeutic responses to nanoparticles [41]. The surface characteristics of nanoparticles influence their *in vivo* fate, and the nano-bio interface—the point at which a nanoparticle interacts with a biological entity, such as a cell, tissue, or biomolecule—is a key regulator of this interaction. Consequently, selecting the right nanomaterials is critical for designing effective nanomedicine carriers.

The immune system recognizes and eliminates foreign substances through multiple mechanisms, with macrophages and dendritic cells (DCs) playing a pivotal role in this process. These immune cells identify and clear exogenous nanoparticles via phagocytosis. Furthermore, they activate adaptive immune responses via antigen presentation, further enhancing nanoparticle clearance. The surface chemistry of nanoparticles can interact with pattern recognition receptors (PRRs) of the complement system, triggering complement activation cascades. This process not only enhances the efficiency of nanoparticle recognition and phagocytosis by immune cells but also leads to rapid nanoparticle clearance. The sustained clearance activity of the immune system typically results in the gradual degradation of nanoparticles within minutes to hours. Furthermore, certain nanoparticles, such as metal oxide nanoparticles, may exhibit pro-inflammatory properties, which can activate macrophages and trigger the release of pro-inflammatory cytokines, including TNF-α and IL-6. The release of these cytokines can induce local or systemic inflammatory responses, potentially causing tissue damage and functional impairments. This inflammatory response not only diminishes the therapeutic efficacy of nanoparticles but also poses potential toxic risks to the body.

In light of the complex immune responses elicited by foreign nanoparticles, researchers have initiated exploring how to leverage these properties to optimize nanoparticle design and their applications. Immunotherapy has emerged as a promising therapeutic modality [42]. Engineered nanoparticles are utilized in tumor nanomedicine to deliver therapeutic agents while evading immune recognition and traversing biological defense barriers. This strategy not only enhances targeted delivery but also improves therapeutic efficacy. Furthermore, these nanoparticles can interact with the immune system to achieve both targeted delivery and immunomodulation.

Although research into the innate immune system remains relatively limited, several immunotherapies that leverage its potential have already received clinical approval [43]. Nanomaterials can interact with various myeloid cells, including neutrophils, monocytes, macrophages, and DCs, by targeting cell surface receptors. Therapeutically modulating the innate immune system can augment adaptive immune responses, presenting potential therapeutic avenues for diseases such as cancer and infections [44]. Conventional nanomedicine designs typically aim to minimize myeloid cells uptake. The physicochemical properties and *in vivo* effects of nanocarriers are determined by their composition. For instance, polyethylene glycol (PEG)-coated liposomes can prolong their circulation time in the bloodstream, thereby increasing drug accumulation at the lesion site. Alternatively, the innate immune system’s affinity for nanomaterials can be leveraged to modulate immune responses [45]. Nanocarriers coated with immunogenic compounds are a standard component of nanomedicine-based immunotherapies. Synthetic analogs, such as lipoprotein and albumin, are used to mimic the natural lipid nanomaterial interactions with the immune system and are commonly used in nanocarrier preparation. Lipoprotein-based nanotherapies have demonstrated significant potential in their interactions with the immune system [46], although the application of synthetic high-density lipoprotein is somewhat confined to selective scenarios.

As previously noted, immunomodulation and treatments for diseases involving phagocytic cells benefit from the immune system's capacity to intercept nanotherapeutics. A deep understanding of the complex mechanisms that govern the interactions between nanotherapeutics and the immune system is crucial, particularly when weighing both the potential benefits and risks. Therefore, drug delivery system design should prioritize avoiding disruption of immune surveillance, as such disruptions could impair immune function and increase susceptibility to external threats [47].

Potential alternative strategies to mitigate immune system interference include the use of extracellular vesicles (EVs) and cell membrane coatings. EVs are naturally occurring nanoparticles secreted by eukaryotic cells, mediating intercellular signaling by transporting lipids, proteins, and nucleic acids. Owing to their low immunogenicity, EVs have been explored as nanocarriers for cancer treatment. Cell membrane-coated nanoparticles, utilizing cell membrane camouflage for efficient therapeutic agent delivery, represent a novel drug delivery strategy [48]. Most current models focus on active targeting to enhance drug delivery, as well as theranostics, aiming to increase drug accumulation in target cells. For example, nanoparticles coated with macrophage membrane-derived vesicles can achieve gradual and precise drug release in response to pH changes in the tumor extracellular microenvironment (TEM). These membrane vesicles retain functional proteins, acting as camouflage that enhances reticuloendothelial system opsonization and promotes the clearance of tumor cells, while also directing nanoparticle accumulation. Nanotherapeutics carrying antiviral agents and coated with neutrophil membranes can bypass detection by macrophages in the spleen and liver, thereby facilitating targeted delivery to sites of inflammation [49]. These complexes not only target inflammation effectively, but also provide longer circulation times and controlled, gradual drug release.

DCs, the most important antigen-presenting cells (APCs) in mammals, play a critical role in innate immunity by recognizing, internalizing, and processing antigens necessary for activating antigen-specific T cells [50]. Consequently, DCs are crucial in cancer immunotherapy. For example, Sipuleucel-T, the only FDA-approved DC-based vaccine for prostate cancer, involves a personalized approach where DC precursors are harvested from each patient and pulsed with a fusion protein of prostate-specific antigen and the cytokine granulocyte-macrophage colony-stimulating factor, which facilitates antigen-presenting cell maturation. However, owing to the presence of immunosuppressive molecules in the TME, tumor-conditioned DCs are often characterized by abnormal lipid accumulation and hyperactivation of the inositol-requiring enzyme 1 alpha/X-box binding protein 1 pathway. This dysregulation, driven by excessive oxidative and ER stress, leads to tumor conditioned DCs dysfunction. Furthermore, intratumoral DCs originate from distinct pathogenic lineages and exhibit diverse functional states. Currently, DNA origami technology is being employed to optimize the spatial distribution of CpG oligodeoxynucleotides to enhance their adjuvant function. Research has demonstrated that precise control over the arrangement of CpG significantly improves DCs activation, antigen presentation efficiency, inflammatory cytokine release, and antigen-specific immune responses. When designed optimally, CpG motifs can trigger robust antigen-specific immune responses and suppress tumor growth [51].

Despite significant advances, the mechanisms underlying nanoparticle-immune system interactions *in vivo* remain poorly understood. Numerous challenges persist, particularly in cases where immune function is compromised or impaired. Nonetheless, recent developments offer optimism that engineered nanoparticles can be rapidly, efficiently, and safely applied in clinical settings.

#### 3.1.3 Targeting ligand and matrix material based on the cancer omics profiles of patients

Research into nanotherapeutics has been actively pursued in cancer theranostics, particularly for treating intermediate and advanced-stage cancers. However, challenges such as low specificity, ineffective targeting, and inadequate accumulation of therapeutic and diagnostic agents at the target site pose significant obstacles to the clinical translation of nanomedicine compared with conventional systemic therapies. Numerous studies have demonstrated that ligand-modified nanocarriers can deliver drugs to specific targets via the EPR effect, with targeting ligands binding to specific receptors on cancer cell surfaces. Unlike normal cells, which typically lack these specific surface markers, tumor cells often present them in abundance [52]. This selective expression makes ligand-directed targeting of cancer cells a crucial strategy for distinguishing between target and non-target cells.

The expression of receptors on cancer cells can be affected by various factors, including patient age, sex, ethnicity, and geographic origin [53]. This variability, coupled with the heterogeneity of cancer cells, poses major challenges to the efficacy of targeted therapies. For instance, the overexpression of receptors, as seen in therapies targeting the estrogen receptor, may lead to mutations like those in ESR1, which in turn impact the tumor sensitivity of metastatic hormone receptor-positive breast cancer [54]. The heterogeneity of ESR1 mutations necessitates molecular monitoring and personalized treatment strategies to mitigate resistance. The expression of chemokine receptors influences the infiltration of various immune cell populations into liver tumors, contributing to heterogeneity [53]. Furthermore, nanotherapeutics are susceptible to chemical and enzymatic degradation. Organic liposomes, for example, are prone to enzymatic breakdown and absorption by the body. Inorganic nanoparticles also undergo both types of metabolism. In the liver, high esterase concentrations render materials like polylactic acid particularly susceptible to hydrolysis, breaking down into glycolic acid and lactate. Alcohol dehydrogenase primarily oxidizes PEG and its derivatives, with CYP450 playing a minor role in this process [55]. Although nanomedicine substrates can be metabolized, targeting ligands such as folate or transferrin are also vulnerable to enzymatic degradation, further complicating the stability and efficacy of nanoparticle-based therapies.

Given these complexities, a personalized approach to nanomedicine is essential, necessitating a deeper understanding of the TME. The emergence of cancer omics-based precision medicine platforms is a direct response to these challenges. Within the precision medicine paradigm, nanomedicines are tailored to a patient’s specific omics profile, representing a significant advancement in the field. The selection of ligands and matrix materials for nanoparticle formulations significantly influences their stability, biodistribution, accumulation, and efficacy, making their optimization a critical challenge in personalized nanomedicine research and application.

In drug discovery, identifying new drug-target interactions is a key step. Accurately predicting binding interactions between chemicals and proteins is crucial for discovering novel therapeutic targets, reducing clinical trial failures, and predicting drug safety. Advances at the intersection of biology and computer science, such as integrating structural data with systems biology to identify novel ligands [56], developing online servers for protein target identification, and combining ligand similarity searches with docking and binding analysis to predict potential targets, have greatly enhanced drug discovery. Computational techniques have also been employed to predict binding conformations and persistent binding sites within enzyme active sites [57]. However, these approaches face challenges such as high computational complexity, long runtimes, and the absence of 3D protein structures, limiting their scalability and feasibility for large-scale testing.

#### 3.1.4 Tumor biology perspectives: key factors in nanomedicine - health, gender, and age

In the vast field of nanomedicine, the majority therapeutic nanomedicine products fail in clinical trials. An in-depth analysis of these failures reveals a key factor: the insufficient consideration of biological variables [58]. Tumor biology provides a unique perspective by focusing not only on the biological basis of disease but also on how individual differences influence therapeutic outcomes. Among the critical factors, health status, sex, and age significantly affect individual responses to nanomedicine.

##### 3.1.4.1 Health status

An individual's overall health, encompassing both physiological and psychological conditions, impacts their tolerance and response to nanomedicine treatments. Pathological conditions, such as chronic diseases or organ dysfunction, can significantly alter drug metabolism, distribution, and excretion, ultimately affecting therapeutic outcomes. For example, individuals with impaired liver function may experience delayed metabolism and clearance of nanomedicines, leading to prolonged drug retention in the body. Similarly, renal dysfunction may impair drug excretion, further contributing to the risk of accumulation and related toxic effects. Furthermore, certain chronic diseases, such as diabetes, cardiovascular diseases, or inflammatory conditions, can affect key physiological factors like hemodynamics and vascular permeability.

Conversely, individuals with optimal overall health are more likely to exhibit enhanced responses and greater tolerance to nanomedicines, owing to the optimal functioning of their physiological systems. Healthy individuals are better equipped to metabolize, distribute, and excrete nanoparticles, resulting in more predictable therapeutic outcomes. Therefore, understanding an individual’s health status and any underlying pathology is crucial when designing and administering nanomedicine-based treatments, as it directly impacts the overall efficacy and safety of these therapies.

##### 3.1.4.2 Gender

Sex-related factors significantly influence the interactions between nanoparticles and biological systems, including cellular uptake, intracellular trafficking pathways, and the composition of biomolecular or protein coronas on nanoparticle surfaces [59]. These differences stem from variations in sex hormone levels, immune system composition, chromosomal inactivation patterns, and physiological characteristics, such as body water distribution. As a result, male and female cells often exhibit distinct responses to nanoparticles, which can impact their pharmacokinetics, circulation time, and immune system interactions.

For example, changes in sex hormone levels in postmenopausal women can alter plasma protein levels, thereby affecting the composition of nanoparticle protein coronas. Sex-specific differences in the mitigation of oxidative stress are observed between male and female fetuses [60], which in turn influence cellular responses to nanoparticles. Additionally, the presence of vitellogenin family proteins in female plasma can alter the biological identity of nanoparticles, leading to notable variations in circulation time, pharmacokinetics, and immune responses to these nanoparticles between males and females [61]. Moreover, variations in cellular structure and function, such as differences in actin fiber arrangement, shape, and distribution, may contribute to sex-specific nanoparticle uptake [59]. Furthermore, disparities in the function and number of immune cells between sexes result in differing immune responses to nanoparticles [62]. The differential inactivation of sex chromosomes may also contribute to varying nanoparticle responses between male and female cells. Female cells exhibit random inactivation of one X chromosome, which may influence nanoparticle interactions differently than male cells. In addition, differences in body water distribution between sexes may affect the biological properties of nanoparticles *in vivo*. Studies have shown that women are 20%–30% more sensitive to water-soluble drugs, such as rocuronium, pancuronium, and vecuronium, compared with men [63].

##### 3.1.4.3 Age

Age-related differences in the antitumor efficacy of cancer nanomedicines have been observed, with biological aging affecting both the clearance and effectiveness of these treatments. Studies in mice have shown that older animals exhibited improved tumor delivery and therapeutic outcomes compared with younger ones. This is likely due to the age-related decline in the capacity of hepatic phagocytic cells to uptake and clear nanoparticles. Specifically, distinct CD11b+ cell subpopulations exist in the livers of young and old mice. Macrophages in younger mice exhibit greater activity in phagocytosis, lysosome function, and antigen presentation pathways [64]. These findings suggest that nanotechnology-based cancer therapies may be less effective in younger patients, underscoring the need for further research on the impact of aging on treatment responses.

Transcriptomic and proteomic analyses reveal that the number of liver macrophages expressing the scavenger receptor MARCO is age-dependent, with a significant reduction in expression in the bone marrow-derived macrophages of aged mice. Therapeutic blockade of MARCO can decrease nanoparticle phagocytosis and enhance the antitumor effects of nanomedicines, particularly in younger mice [65].

Biological variables play a critical role in shaping the mechanisms of action and therapeutic responses of nanoparticles. Health status, which reflects an individual's overall physiological and psychological condition, determines the tolerance and response to treatment. Sex differences influence drug metabolism, disease susceptibility, and treatment outcomes, whereas age is closely associated with biomarker expression, pharmacokinetics, and pharmacodynamics. These factors collectively shape personalized nanomedicine treatment strategies. Therefore, considering multiple biological variables is essential for optimizing therapeutic strategies and improving patient outcomes. Researchers and clinicians in nanomedicine must prioritize these factors in study design and implementation to ensure effective and individualized patient care.

### 3.2 Which extravasation mechanism is more important?

Extravasation refers to the process by which nanoparticles or other substances pass through the vascular wall and enter tumor tissue via the EPR effect, a critical step in passively targeting cancer cells with nanomedicine. Although the exact mechanisms of extravasation are not yet fully understood, several factors influence this process. These factors include nanoparticle size, shape, surface charge, coating, as well as tumor vasculature characteristics, such as pore size, permeability, and the expression of adhesion molecules.

Nanoparticles extravasate into tumors primarily through two pathways: paracellular and transcellular [66]. The paracellular pathway involves the movement of nanoparticles through gaps between endothelial cells, whereas the transcellular pathway involves nanoparticles passing directly through endothelial cells. The selection of these pathways may be influenced by nanoparticle properties and the TME. Therefore, a comprehensive understanding of extravasation mechanisms is essential for optimizing the tumor-targeted delivery of nanomedicines.

#### 3.2.1 Heterogeneity of the EPR effect

**Fig. 3:**
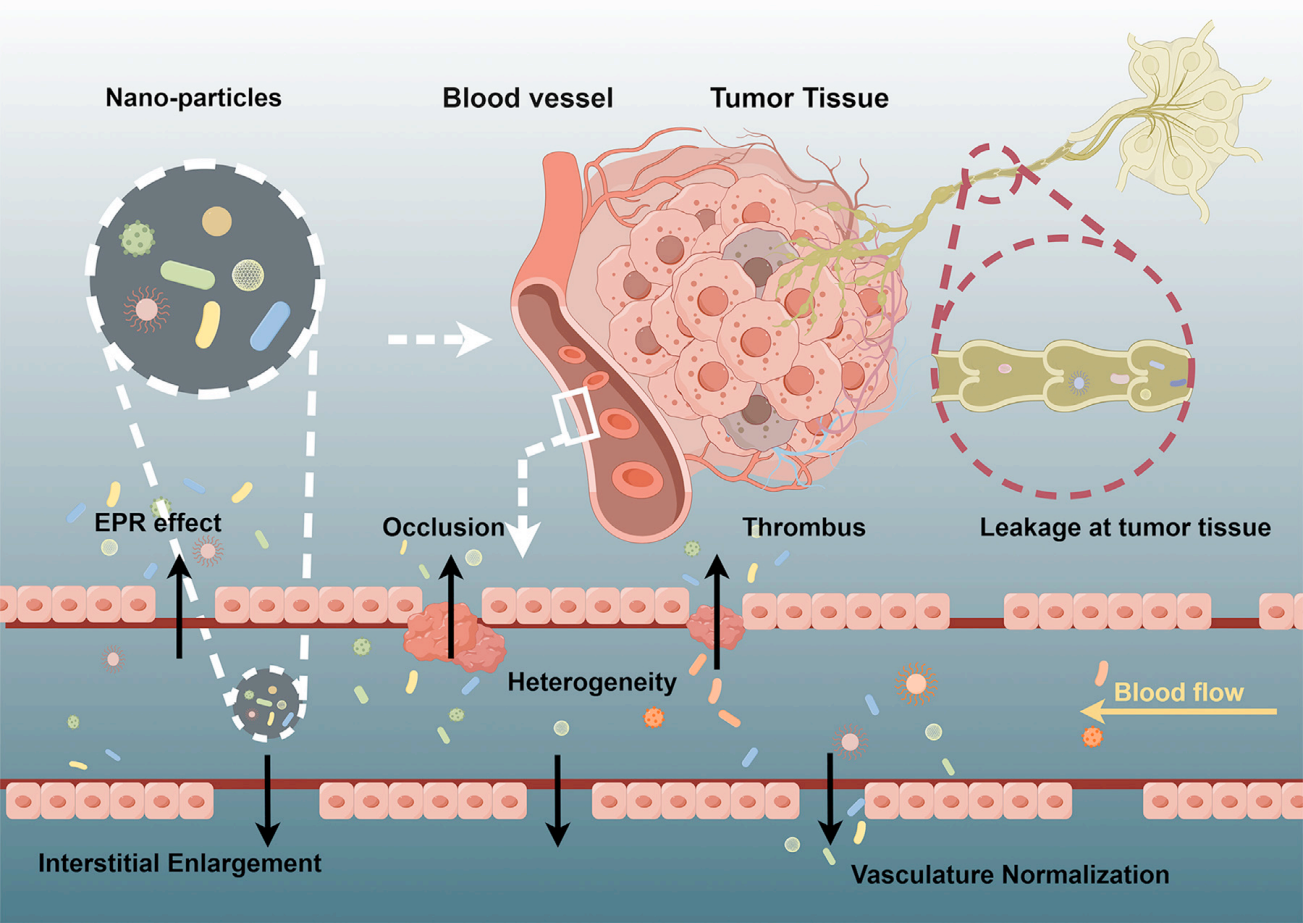
**Limitations in the continuous transportation of nanomedicine within tumors.** The post-entry continuous transport pathway of nanomedicine in the body, and the encountered EPR heterogeneity and lymph node drainage. (Arrows denote the direction of nanoparticle movement).

Nanoparticle accumulation within tumors is a complex process driven by a broader range of biological mechanisms than previously understood. These mechanisms encompass angiogenesis, hemodynamic regulation, vascular permeability, lymph angiogenesis, and the intrinsic heterogeneity of the TME, as depicted in Fig 3. Notably, tumors exhibit impaired lymphatic drainage, leading to decreased interstitial fluid absorption compared with normal tissues [67]. This impaired drainage, along with the diffusion barriers that nanoparticles encounter due to their hydrodynamic radius, results in the preferential accumulation of larger nanoparticles within the tumor interstitial. In contrast, molecules smaller than 4 nm are rapidly reabsorbed and returned to the bloodstream [68].

Nanoparticles leverage the unique tumor vasculature and EPR effect to mitigate drug degradation, prolong circulation times, and facilitate preferential accumulation within tumors, thereby increasing local drug concentrations. After extravasation, the nanoparticle accumulation *in situ* is further amplified by impaired lymphatic drainage. This passive tumor-targeting strategy enhances the therapeutic indices of chemotherapeutic agents and substantially reduces systemic toxicity compared with conventional drug delivery systems.

The therapeutic efficacy of first-generation anticancer nanomedicines largely depends on the EPR effect, which, however, varies due to the inherent heterogeneity in tumor vascular architecture and permeability. Consequently, passive targeting strategies that rely solely on the EPR effect may lack uniform efficacy and lead to inconsistent clinical outcomes, particularly in metastatic cancers with heterogeneous tumor vasculature. The EPR effect is associated with distinct anatomical and pathophysiological features of solid tumors, including aberrant vasculature, increased intercellular permeability, and elevated levels of vascular mediators. These features contribute to significant extravasation of plasma components and subsequent nanodrug accumulation. The following section will elaborate on the heterogeneous nature of the EPR effect in more detail.

##### 3.2.1.1 Vascular permeability

Multiple factors influence the kinetics and heterogeneity of the EPR effect, including pathophysiological and anatomical characteristics, drug formulation, and physicochemical properties [69]. Tumor tissues often exhibit heterogeneous vascular densities and permeabilities. The rapid proliferation of tumor cells necessitates an augmented blood supply, driving accelerated angiogenesis and contributing to this heterogeneity. These processes result in the formation of irregular vascular architectures characterized by discontinuous endothelial layers and the absence of a basement membrane. As a result, leaky vessels with pore sizes ranging from 200 to 2000 nm arise [70], ultimately influencing the ability of nanoparticles or drug carriers to penetrate tumor tissue.

##### 3.2.1.2 Interstitial pressure

Elevated interstitial fluid pressure (IFP), which is a result of increased vascular permeability, constitutes a significant barrier to the convective diffusion of nanoparticles [71]. The transport efficiency of nanoparticles declines as IFP rises. When IFP approaches the pressure within the blood vessel wall, the ability of nanoparticles to extravasate into the tumor interstitial is blocked. The elimination of the pressure gradient within the tumor effectively negates the EPR effect, hindering nanoparticle delivery. Significant inter- and intra-tumoral heterogeneity in the EPR effect is well-documented, with variations observed across patients, various tumor types, different tumor regions, and even within the same tumor over time.

##### 3.2.1.3 Normalization of tumor vessels

In the course of rapid tumor growth, increased vascular permeability facilitates the delivery of nutrients and oxygen necessary to sustain the accelerated growth rate. Furthermore, certain therapeutic interventions can induce tumor vessel normalization, thereby altering vascular permeability and structure and influencing the EPR effect [72].

As elaborated earlier, the EPR effect displays inherent heterogeneity throughout tumor progression, with variations observed across diverse stages of disease development. This inherent heterogeneity in the EPR effect has profound implications for drug pharmacokinetics, biodistribution, and ultimately, therapeutic efficacy, posing significant challenges for the design and evaluation of effective nanomedicine-based therapeutic strategies.

#### 3.2.2 Continuous nanomedicine transportation, basement membrane permeation and lymphatic drainage in tumor

Despite extensive research in recent decades, the clinical translation of nanomedicines for anticancer therapy remains limited. One of the main factors accounting for this limited translation is the inherently low tumor permeability of numerous nanomedicines. Specifically, achieving efficient and sustained transport, basement membrane permeation, and modulation of lymphatic drainage are all critical yet challenging aspects of nanomedicine design for effective tumor treatment.

##### 3.2.2.1 Limitations of basement membrane permeation

The basement membrane of tumor vasculature acts as a substantial mechanical barrier, hindering the effective penetration of nanotherapeutics. This barrier significantly impacts nanomedicine delivery by restricting extravasated nanoparticles to the perivascular region and preventing their penetration beyond the subendothelial space [40]. Consequently, the efficacy of nanotherapeutic delivery in the context of tumor treatment is influenced by the complex interplay between the TME, the physicochemical properties of the nanomedicine, and the barrier function of the tumor vascular basement membrane. Furthermore, it is crucial for nanomedicines to exhibit selective permeability across the basement membrane to target tumor cells while minimizing off-target effects on normal tissues. These challenges highlight the critical need to carefully consider and optimize the interplay between nanomedicine design, TME characteristics, and basement membrane properties to achieve effective tumor targeting and improved therapeutic outcomes. Future research should prioritize developing nanomedicines with enhanced basement membrane penetration and tumor cell selectivity, ultimately improving the efficacy of nanomedicine-based cancer therapies.

##### 3.2.2.2 Limitations of lymphatic system drainage

Nanoparticles designed based on the EPR effect frequently fail in clinical trials, potentially due to an insufficient understanding of the mechanisms underlying the delivery process. These nanoparticles passively infiltrate tumors through endothelial cell gaps and are retained due to impaired lymphatic drainage, which can trigger adverse effects such as immunosuppression, fibrosis, foreign body reactions, oxidative stress, and DNA damage. Excessive retention of nanoparticles within tumors can also impair their secondary circulation capacity, thereby reducing their overall utilization in the body. Moreover, nanomedicine delivery systems may exhibit off-target effects, wherein drugs may be redirected to non-target areas, reducing therapeutic efficacy and potentially causing unnecessary side effects.

A recent study has emphasized that the Active Transport and Retention (ATR) principle challenges the traditional EPR effect [73], proposing that nanoparticles gain entry into tumors via an active endothelial transport mechanism, interact with tumor components, and are then retained and drained through the lymphatic system. This suggests that nanoparticle delivery relies not only on passive permeability but also involves active cellular processes. Research indicates that a portion of nanoparticles entering tumors can still be drained via the lymphatic system and re-enter the bloodstream, and this dynamic delivery can help improve therapeutic efficacy.

The complexity and heterogeneity of the lymphatic system are particularly evident in different types of tumors, leading to suboptimal drainage of nanomedicines in some tumors, thereby impacting therapeutic efficacy [74]. Lymphatic drainage speed varies by tumor location, with faster rates in distal limbs and slower rates in the head, neck, and proximal limbs; additionally, lymphatic drainage plays a crucial role in tumor development, immune response, and metastasis. Precise control of nanomedicine release at specific times and locations is a significant technical challenge; moreover, the dynamic nature of the lymphatic system further complicates this issue. Despite the significant potential of nanomedicine delivery systems demonstrated in laboratory and animal models, their performance may not translate effectively to clinical trials. The limited number of clinical trials and insufficient data hinder a comprehensive understanding.

These challenges emphasize the necessity of comprehensively considering the complexity of the TME and the physicochemical properties of nanomedicines during the design process. Researchers are developing novel nanomedicine platforms to address these challenges, including enhancements in stability, targeting, permeation, and controlled release. Moreover, advancements in biomaterials and nanotechnology are facilitating the development of innovative strategies to improve the effectiveness of nanomedicines in tumor treatment. Comprehensive research is expected to yield safer and more effective tumor treatments.

### 3.3 How to manufacture precise nanomedicine?

Nanomedicine, as an emerging field, holds the potential to revolutionize medical practice and improve patients’ quality of life. It embodies essential characteristics for future nanomedicines, including multifunctionality, intelligence, precision, and personalization. A major challenge in nanomedicine is the absence of standardized methodologies for characterizing, evaluating, and regulating nanomedicines, which hinder their translation from the laboratory to clinical practice. Furthermore, the ethical, social, and legal implications of nanotechnology necessitate careful consideration and effective communication with the public and stakeholders. To tackle these challenges, current nanomedicine research is focusing on several key directions. First, it involves redesigning nanomedicines to optimize their design, surface modification, targeting, and stimulus responsiveness, aiming to enhance tumor specificity and minimize unnecessary interactions. Second, leveraging the nanoparticle biological barriers and interactions to develop novel diagnostic and therapeutic applications, such as imaging, biosensing, immunotherapy, and gene therapy. Third, employing advanced tools and models to gain deeper insights into nano-bio interactions and nanomedicine mechanisms of action. Fourth, establishing multidisciplinary collaboration platforms to promote the exchange of knowledge, data, and resources among researchers, clinicians, regulators, and industry. Fifth, engaging with the public and stakeholders to address concerns, expectations, and needs while fostering responsible and ethical development and utilization. Consequently, nanomedicine necessitates further research, development, and cross-disciplinary collaboration among various stakeholders to ensure its safety, efficacy, and accessibility.

## 4 Future priority focus areas in nano-oncology research

### 4.1 Dynamically integrating multistage tumor-targeting strategies

For effective treatment, nanoparticles must accumulate within tumor tissue and subsequently penetrate deeper into tumor cells and organelles. However, static and unchanging nanoparticles are unlikely to yield satisfactory results at every stage of treatment. This is primarily due to the varying, and at times opposing, physiological characteristics exhibited by different parts of the body. Therefore, integrating dynamic multi-stage tumor targeting strategies is essential.

Generally, nanoparticles must maintain stability during circulation, accumulate in tumor tissue, escape from the endosome/lysosome into the cytosol of cancer cells, and infiltrate various organelles to exert therapeutic effects [75]. However, each of these stages requires the nanoparticle delivery system to possess entirely different surface properties. The optimal nanoparticle size for EPR-mediated tumor accumulation is around 50–200 nm, which, although effective for accumulation, limits deeper tumor penetration [76]. To address this, researchers aim to design nanoparticles capable of shrinking to 10–20 nm after tumor accumulation and further reducing in size for penetration through nuclear pore complexes.

Exploiting characteristic pH differences within the TME can stimulate electrostatic interactions between nanoparticles, enabling them to switch between attraction and repulsion, thereby inducing size changes. Enzymes highly expressed in the TME, such as MMP-2 and hyaluronidase, can also promote nanoparticle size reduction [77].

Regarding charge, nanoparticles with different charges exhibit distinct drawbacks. For instance, positively charged nanoparticles are rapidly cleared from the body, but they are readily taken up by cells and can effectively escape the endosome. In contrast, neutral and negatively charged nanocarriers exhibit longer half-lives and improved tumor accumulation but encounter challenges in cellular uptake. Consequently, developing nanoparticles with charge conversion—exhibiting opposite charges in different environments—represents a promising strategy [78].

Furthermore, the necessary targeting ligands on nanocarrier surfaces can readily bind to blood components or immune cells during circulation, reducing their targeting effectiveness. To overcome these challenges, a strategy has been proposed where the ligands are “shielded” or hidden during circulation and are only exposed or activated upon reaching the tumor site [79].

These nanoparticles typically require endogenous or exogenous stimuli for functional conversion, enabling drug release within a precise therapeutic window, thereby improving efficacy and mitigating side effects. This delivery system enhances the therapeutic index of drugs and provides a novel tool for precision medicine, potentially playing a crucial role in future clinical treatments.

However, integrating these diverse strategies into a single nanomaterial is complex and requires extensive experimental research and meticulous engineering. By integrating this process with AI technology, which excels in data processing and pattern recognition capabilities, a new and promising research avenue could emerge. This integration has the potential to greatly enhance the efficiency and precision of nanocarrier design.

### 4.2 Interdisciplinary fusion drives innovative development of tumor nanotherapeutic strategies

With accelerating scientific and technological progress, the field of tumor nanotherapy is shifting toward interdisciplinary integration. Traditional disciplines such as materials science, biomedicine, and imaging are closely linked to tumor nanotherapeutics. The rapid development of AI has led to innovative breakthroughs, broadening research horizons and application potential.

**Fig. 4:**
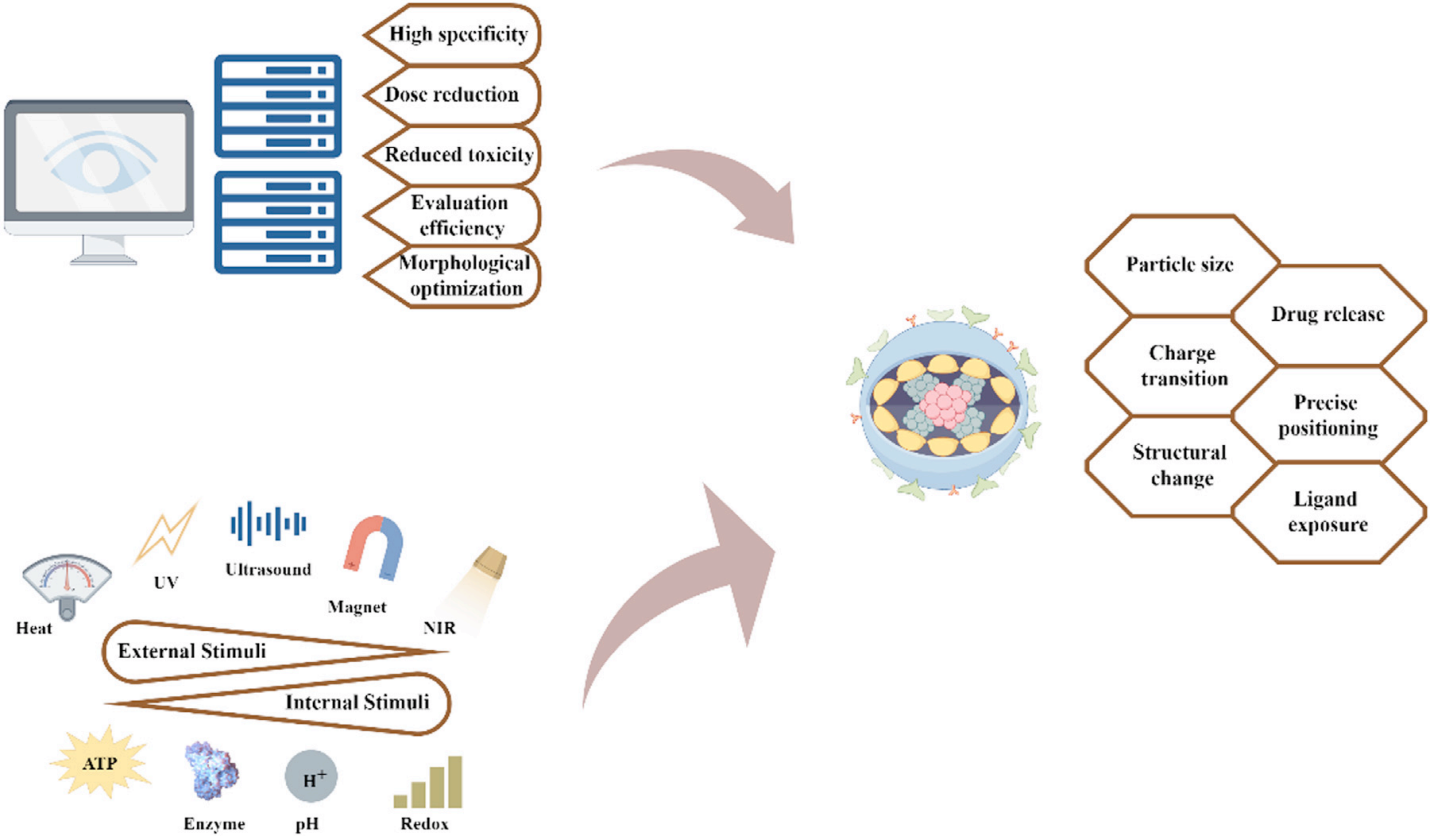
**Computers are utilized to design more effective nanomaterials that can trigger nanoparticle size reduction, charge conversion, and ligand exposure through various stimuli, enabling dynamic integration with tumor tissues and specific targeting of cells and organelles in multi-stage tumor targeting**.

Researchers have found that employing AI to advance nanoparticle research and development holds great promise, particularly in precision therapy. Typically, before employing AI algorithms, researchers must conduct accurate biomarker analysis of patient tumors using omics or nanosensor technologies to identify suitable candidates for clinically targeted therapeutic agents (Fig. 4). Subsequently, the AI-assisted design of suitable nanoparticles enables the intelligent navigation of the complex TME, selective targeting of cancer cells, and more precise therapeutic payload delivery. Additionally, machine learning (ML), a branch of AI, can classify tumors more efficiently, assisting researchers in selecting ideal nanoparticle carriers and improving nanoparticles through large-scale AI analysis for more precise treatments [80].

The integration of AI into tumor nanotherapy is a current trend. Existing public databases and rich experimental data accumulated over years in large laboratories provide sufficient support for AI-driven analysis to extract various features. Such features include nanoparticle size, charge, shape, and composition, as well as cancer subcellular classification, state, and surface receptors, which are critical for predictive modeling and nanoparticle development. This analysis enhances the alignment of simulation results with real-world conditions, reduces the discrepancy between prediction and reality, significantly improves research efficiency, and reduces researcher workload through automated and precise data processing. In addition, accurate simulation results help reduce the need for animal testing, meet ethical standards and speed up the research process [81].

The solvent-accessible surface area (SASA) of nanoparticles is critical for their interaction with the biological environment and subsequent drug delivery efficiency. Rao developed a three-stage ML approach that dramatically improves SASA predictive efficiency, overcoming the computationally intensive, time-consuming, and costly challenges of traditional molecular dynamics simulations. This method improves the accuracy of prediction through training and optimization of ML models, as well as data augmentation techniques. The results demonstrate that applying AI technology has increased prediction speed 40-fold, improved accuracy by 25%, significantly shortened nanoparticle design cycles, and reduced costs, highlighting its immense potential to drive nanoparticle innovation and precision medicine [82]. Akhtar leverages ML to optimize drug delivery pathways, utilizing cardiovascular dynamics data to accurately simulate the movement and deposition of nanoparticles within the body. By analyzing fluid dynamics and predicting optimal pathways, ML algorithms enhance the accuracy of nanoparticle delivery to tumors, mitigating side effects and improving efficacy. Concurrently, the application of magnetic fields is being explored as a complementary strategy to guide nanoparticles directly to cancer tissue. The magnetic field manipulates the distribution of magnetic nanoparticles, ensuring their effective accumulation within the target area. The ML model analyzes the interplay between magnetic force and blood flow to optimize magnetic field parameters. The integration of ML with magnetic field control yields a highly adaptive nanoparticle drug delivery system that offers novel strategies for more precise, personalized, and efficient cancer treatment, demonstrating immense potential within the field of nanoparticle innovation and precision medicine.

In targeted nanoparticle delivery, AI can integrate sensors and imaging agents into nanoparticle systems or utilize advanced equipment, such as high-resolution microscopes, for real-time identification and tracking, enabling multi-stage observation of tumor site changes and characteristics. This approach reveals *in vivo* drug delivery mechanisms, evaluates efficiency, identifies administration obstacles, adjusts doses, and enables intelligent optimization.

For example, Lutz's team employed accurate, reliable, and adjustable ferritin nanocages as fluorescent probes in mice bearing 32 types of subcutaneous tumors, collecting over 67,000 tumor blood vessel fluorescence images. By leveraging ML techniques for image segmentation, a high-throughput AI-assisted classification method was established to rapidly assess tumor vascular permeability and uncover significant heterogeneity among tumors. These results enabled the development of a customized protein nanoparticle system capable of effectively crossing the tumor endothelial barrier and reaching the tumor core. It can be seen that artificial intelligence can assist modeling to optimize the structure of nanoparticles and predict delivery routes, thereby helping to improve tumor penetration, reduce side effects, and improve therapeutic efficacy [83].

However, it is important to recognize that AI-powered nanoparticles remain in the early stages of development for cancer treatment and face numerous hurdles. Developing nanoparticle-based AI models for cancer treatment necessitates extensive datasets. Currently, these datasets are often limited to specific nanoparticle families and lack sufficient characterization. Ensuring the safety and biocompatibility of nanoparticles is crucial. Traditional safety assessments may not be suitable for these novel materials, necessitating the development of new protocols and standards. Clear guidelines and standards are essential to ensureing the safe and effective clinical use of these technologies.

Furthermore, developing efficient and advanced analytical tools is necessary to better understand how nanoparticles interact with biological systems and to monitor their *in vivo* behavior. The absence of *in vivo* monitoring systems hinders the tracking of nanoparticle distribution and efficacy. Gaps remain in our understanding of how nanoparticles interact with biological systems, potentially impacting their safety and effectiveness.

Addressing these challenges is critical for successfully integrating AI and nanotechnology into cancer treatment, ultimately paving the way for more effective and personalized therapies [80].

### 4.3 The personalized perspective of nanomedicine

Personalized cancer medicine is currently a prominent area of research. It is centered on developing customized treatment plans that are tailored to the unique characteristics of individual patients or specific patient subgroups. Studies have demonstrated significant inter-patient variability in the same cancer types, leading to inconsistent and frequently suboptimal treatment outcomes. Moreover, intra-tumor heterogeneity poses a significant challenge, marked by considerable variations in the genetic makeup of cancer cells across different regions within a single tumor [84]. In cancer treatment, multiple factors must be considered, such as age, gender, lifestyle, environmental exposures, individual genomic differences, and treatment responses. The unique properties of nanoparticles facilitate the customization of treatments based on a patient's genetic profile, disease characteristics, and drug response, driving exponential growth in the application of personalized therapy. Personalized nanotherapy utilizes a patient's unique biological characteristics, such as genomics or proteomics, for both therapeutic and diagnostic purposes, employing tailored protocols to optimize treatment outcomes.

Cancer heterogeneity is characterized by the presence of multiple molecular subtypes within a single cancer type, each exhibiting distinct molecular characteristics, pathological features, and treatment responses. This heterogeneity extends beyond the histological level, encompassing the genome, transcriptome, proteome, and other molecular levels. Consequently, the traditional “one-size-fits-all” treatment strategy is often inadequate in addressing the diverse treatment needs of patients with different tumor subtypes. Different tumor subtypes exhibit varying degrees of sensitivity or tolerance to chemotherapy, targeted therapy, immunotherapy, and other therapeutic modalities [85]. For instance, in breast cancer, different molecular subtypes, such as HER2-positive, hormone receptor-positive, and triple-negative breast cancer, exhibit distinct therapeutic sensitivities. HER2-positive subtypes respond favorably to HER2-targeted therapies, while hormone receptor-positive subtypes are typically sensitive to endocrine therapy. In contrast, triple-negative breast cancer lacks a clearly defined therapeutic target and, despite demonstrating sensitivity to chemotherapy, still lacks effective targeted therapy strategies. These differences underscore the necessity for tailored treatment regimens for different molecular subtypes. Pan-cancer studies have focused on KRAS gene mutations, particularly in pancreatic ductal adenocarcinoma, colorectal adenocarcinoma, and lung adenocarcinoma, where KRAS is the most frequently mutated RAS isoform of the RAS family. Li et al. developed a nanotherapeutic strategy targeting the metabolic vulnerabilities associated with KRAS mutations to enhance the precision and efficacy of treatment. By enhancing drug accumulation within tumor tissues, this strategy improves therapeutic efficacy while reducing systemic side effects, underscoring the pivotal role of personalized therapy targeting metabolic pathways in optimizing the treatment of KRAS-driven malignancies. To mitigate the impact of cancer heterogeneity on personalized cancer treatment, the utilization of autologous materials represents an effective strategy. Central to this approach is the utilization of patient-derived biomaterials to minimize variability in treatment response due to cancer phenotypic differences, thereby enhancing treatment consistency and efficacy. For instance, by conjugating fluoropolymers to surgically resected autologous tumor cell membranes, Xu et al. promoted dendritic cell maturation and antigen cross-presentation via toll-like receptor 4 signaling pathways, facilitating the development of effective personalized cancer vaccines. This fluoropolymer-based personalized nanovaccine, in combination with immune checkpoint blockers, can effectively prevent tumor recurrence and metastasis post-surgery [86].

Sex differences play a crucial role in cancer initiation, progression, and treatment response, which can be attributed in part to differences in XY chromosome genetic metabolism that elucidate sex-specific mechanisms of gene expression regulation. Therefore, the development of personalized treatment strategies that consider sex differences is of paramount importance. In the context of immunotherapy, women may have a more significant immune defense mechanism due to random inactivation of the X chromosome, resulting in genetic and epigenetic mosaicism [87]. Additionally, sex differences exist in the toxicity of nanomaterials. For example, gold nanoparticles coated with PEG may induce more severe renal damage in female mice while exhibiting greater hepatic toxicity in male mice [88]. Sex-based differences also exist in the pharmacodynamics and pharmacokinetics of drugs. For example, the uptake rate of quantum dots in female human amniotic stem cells is higher than that in male cells. However, the uptake rate of the same nanoparticles is lower in female salivary gland primary cells compared to that in male cells [59]. These sex-based differences underscore the importance of considering sex as a biological variable in the development of nanomaterial-based cancer therapies, thereby enabling the development of more precise and personalized treatment strategies.

Across multiple domains of cancer research, including genomics, transcriptomics, epigenetics, and immunology, there are substantial biological differences between younger and older patients. While the incidence and mortality of most cancer types increase with age, the incidence of lung adenocarcinoma and endometrial cancer is also increasing among younger age groups. Specific cancer driver gene mutations exhibit varying frequencies in younger and older patients. With advancing age, declining immune function, coupled with the accumulation of chronic inflammation, may promote cancer development. The release of inflammatory cytokines, chemokines, and growth factors, along with alterations in the tissue microenvironment, are thought to drive cancer development and progression. Age-related disruptions in circadian rhythms are also thought to elevate the risk of several cancers [89]. For example, the expression of circadian rhythm genes, including CRY1, CRY2, ROR, and BMAL1, has been associated with prostate cancer progression. Similarly, genes from the PER and CRY families may play a role in the pathogenesis of lung adenocarcinoma, making them potential targets for personalized therapy [90].

Additionally, elevated claudin-1 expression, particularly during aging, has been implicated in compromised blood-brain barrier function. In response, Badrul developed a claudin-1-targeting nanoparticle that specifically binds to purified claudin-1, enabling efficient accumulation and retention in the cerebrovascular system. This targeted approach facilitates nanoparticle delivery to brain regions with impaired functionality. This design strategy offers a novel approach for nanomedicine delivery systems in brain cancer therapy [91].

Pediatric cancer, although relatively rare, presents unique challenges due to the involvement of smaller organs and more severe long-term consequences of treatment. The distinctions between childhood and adult cancers remain largely unexplored, and the potential long-term effects of existing treatments are significantly greater in children, necessitating tailored approaches [92]. Moreover, studies indicate that factors such as cigarette smoke, alcohol consumption, air pollution, contaminated water or food, exposure to contaminated soil, ionizing radiation, sleep deprivation, and obesity are strongly linked to human cancer, resulting in variations in cancer characteristics in affected patients [93]. For example, one study utilizing deep neural networks found that smoking frequency was a significant risk factor for lung cancer in men over 65. Prompt smoking cessation can reduce the risk of lung cancer mortality later in life [94]. These factors also provide potential targets for personalized treatment.

However, identifying reliable and specific biomarkers for various diseases and subtypes is a complex and costly process that necessitates extensive data analysis and validation. Additionally, developing nanomedicine detectable by various imaging modalities presents challenges that necessitate complex engineering and integration of multiple components. Regarding safety considerations, the significant variability in diseases and patients, coupled with the complex interactions between nanoparticles and biological systems, makes predicting and evaluating therapeutic safety and efficacy in personalized medicine more intricate and challenging [95]. Another significant challenge is the economic burden of personalized nanomedicine. It is costly to develop, requires years of funding, and has a low probability of success. The technology required for DNA sequencing and nanomedicine design further raises these costs. In cancer therapy, the broad range of genetic mutation targets presents opportunities for personalized nanotherapy but also contributes to increased costs.

Customized and convenient treatment options can enhance patient compliance and satisfaction. Personalized nanomedicine is a promising emerging field with the potential to transform cancer treatment and improve patient outcomes across various diseases. Now is the time to embrace this challenge and revolutionize cancer medicine through nanotherapeutics.

### 4.4 Precise nanomanufacturing technology

With the continuous development of nanomedicine, researchers have established more rigorous standards for the preparation and modification of nanomaterials. This demand has led to the development of precision nanomanufacturing technologies, which enable the precise synthesis and regulation of nanomaterials at the atomic and molecular levels [96]. Through meticulous design, customization, and optimization of the structure and function of nanomaterials, researchers can rigorously analyze the structure-activity relationship between the structural properties and functional characteristics of materials. This approach opens avenues for new functions and expands the application prospects for the research and development of nanomedicine.

Microfluidic technology is being increasingly employed in precision nanomanufacturing; however, its design heavily relies on researcher expertise and computational simulations. The complexity of multiphase flows, inertial processes, and performance indicators involving biological and chemical parameters (*e.g.,* synthetic yield, expression variation, and morphology) makes it challenging for computational methods to accurately describe the behavior of these intricate systems [97]. Limitations in equipment design and operational parameters also complicate precise dimensional control. Moreover, maintaining stable operating conditions, such as temperature, pressure and pH, further poses challenges to the achievement of reliable and reproducible results.

A current trend is the integration of AI with microfluidics, leveraging its data analysis and predictive capabilities to achieve intelligent and precise synthesis of nanomaterials. Additionally, the convergence of 3D printing technology with precision medicine is becoming increasingly significant. Extrusion 3D printing (including FDM and PAM) is a key technology for fabricating solid dosage forms, enabling the transformation of nanocarrier drug delivery systems into stable solid forms while preserving the nanomaterial properties of the drugs. The combination of 3D printing and microfluidic chips significantly advances the development of precision nanomedicine. Nevertheless, challenges such as the complexity of 3D printing, inadequate regulation frameworks, lack of operational training, and contamination issues limit its clinical application. To accelerate clinical translation, it is essential to enhance the resolution, complexity, repeatability, and speed of 3D printing and biomanufacturing technologies. Additionally, expanding the range of materials, including biological materials such as cells, and ensuring robust regulatory documentation are vital for success [98].

DNA origami technology represents an advanced nanomanufacturing method that enables the precise design of two-dimensional and three-dimensional structures at the nanoscale. Its capabilities in precise structural control, functionalization, biocompatibility, and intelligent design render it critical for drug delivery, targeted therapy, and the construction of multifunctional nanoplatforms. The integration of AI further enhances the entire DNA origami process, from design and synthesis to functionalization, screening, and *in vivo* monitoring. This interdisciplinary collaboration clears the path for a broad array of applications of DNA-based nanomaterials in the biomedical field [99].

In summary, the development of precision nanomanufacturing technology can significantly advance nanomedicine research and foster the integration of multidisciplinary technologies. The application of these advanced methods offers new ideas and strategies for nanomedicine design and preparation, with the potential to overcome existing challenges and realize precise therapies. Looking ahead, as technology continues to progress and interdisciplinary cooperation deepens, there is ample reason to believe that nanomedicine will find broader applications in personalized medicine, disease diagnosis, and treatment, ultimately bringing revolutionary changes to human health.

## 5 Conclusion and prospects

Nano-oncology has achieved remarkable progress since its emergence, and it utilizes nanotechnology to improve cancer diagnosis, treatment, and monitoring. The advancements in selective targeting-based nanomedicines, which can precisely deliver therapeutic agents at multiple levels, ranging from tissues to organelles, along with smart nano-systems that respond to tumor-specific conditions for controlled drug release and reduced systemic toxicity, and multidrug nanomedicines that overcomes drug resistance by co-delivering multiple agents, illustrate the field's potential to revolutionize cancer therapy. These innovations are designed to enhance the specificity and efficacy of treatments while minimizing adverse effects, thereby improving patient outcomes.

Nevertheless, numerous challenges remain to be addressed to realize the potential of nano-oncology. Key issues, such as the accumulation of nanoparticles in tumors, the interaction between nanoparticles and the immune system, and the heterogeneity of the EPR effect, pose significant obstacles. Furthermore, advancements in precise nanomanufacturing and the synthesis of novel nanomaterials are essential for overcoming current limitations. Innovations in nanomanufacturing can enhance the scalability, reproducibility, and cost-effectiveness of nanomedicines. Novel nanomaterials with improved properties, such as enhanced biocompatibility, stability, and targeting capabilities, can further augment the therapeutic potential of nanomedicines.

Looking ahead, the future of tumor nanotherapy is inextricably linked with advancements in personalized medicine. The essence of personalized therapy is to tailor precise and targeted cancer therapies and treatment plans based on individual patient characteristics, such as genetic profiles and tumor biomarkers. This approach aims to maximize therapeutic efficacy while minimizing side effects and to predict treatment outcomes for timely adjustments as needed. Undoubtedly, this requirement increases the complexity and challenge of implementing the technology. It demands a deep understanding of the properties of nanomaterials and an accurate grasp of patient pathological features to ensure the precision and effectiveness of treatment plans. Paying attention to details and technological refinement is crucial for achieving the goals of personalized medicine.

AI is anticipated to play a pivotal role in promoting tumor nanotherapy. By analyzing extensive patient data, AI can optimize treatment protocols and predict therapeutic outcomes. Machine learning algorithms can assist in the design of novel nanomaterials with enhanced drug delivery capabilities and improved biocompatibility, thereby accelerating the development of next-generation therapies. AI has expedited the novel drug discovery by predicting the interactions of various compounds with cancer cells at the nanoscale. This speeds up the identification of promising drug candidates and reduces the time and costs associated with bringing new therapies to market. Moreover, the discovery and integration of novel nanomaterials hold significant promise for augmenting the therapeutic arsenal available for tumor nanotherapy. From biodegradable nanoparticles capable of sustained drug release to multifunctional nanocarriers that integrate therapeutic and diagnostic functions, these advancements are poised to revolutionize cancer treatment.

In summary, the future landscape of tumor nanotherapy envisions a convergence of personalized medicine, AI-driven innovations, and the development of novel nanomaterials. These synergistic advancements possess immense potential to transform cancer care by providing more effective, targeted, and patient-centered therapeutic approaches.

## Declaration of competing interest

The authors declare that they have no conflicts of interest in this work.

## Appendix Supplementary materials

Image, application 1
