# Supplementary material for: Nano-oncology revisited: Insights on precise therapeutic advances and challenges in tumor
Source: Fundam Res. 2025 May 14;5(5):1845–59. doi: 10.1016/j.fmre.2025.03.024 (PMC12848196; doi:10.1016/j.fmre.2025.03.024)
Supplement: Supplementary file 1 [file mmc1.docx]

**Table S1. Recent progress of nanomedicine in cancer treatment and diagnosis**

|  | **Nanotechnology platform** | **Nanomaterials** | **Active pharmaceutical ingredients** | **Therapy modality** | **Mechanism of Action** | **Cancer type** | **Ref** |
| --- | --- | --- | --- | --- | --- | --- | --- |
| **Organic Materials** | Lipid-Based Nanomaterials- Liposomes | Dual-Targeting Liposome | Paclitaxel (PTX) and Danshensu | Chemoimmunotherapy | Inducing Immunogenic Cell Death (ICD) and inhibiting STAT3 activation | Triple-Negative Breast Cancer | [1] |
|  |  | Sequential Targeting Hybrid Nanovesicles | PTX | Chemoimmunotherapy | Sequential targeted delivery, blocking PD-L1 to avoid T cell exhaustion, promoting PTX-induced immunogenic cell death | Lung Cancer | [2] |
|  |  | Combinatorial Polydopamine-Liposome Nanoformulation | Polydopamine, Doxorubicin (DOX) and Indocyanine Green | Chemotherapy & laser-induced Photodynamic Therapy (PDT)/Photothermal Therapy (PTT) | Co-delivery of drugs using polydopamine-coated liposomes, generating reactive oxygen species (ROS) for PDT, and PTT with laser activation | Breast Cancer | [3] |
|  |  | Dual-Ligand-Functionalized Liposomes | DOX | Chemotherapy | Targeting hepatocellular carcinoma with dual-ligand-functionalized liposomes using Glycyrrhetinic Acid and cRGD. | Hepatocellular Carcinoma. | [4] |
|  | Lipid-Based Nanomaterials-Solid Lipid Nanoparticles | Dacarbazine-Encapsulated Solid Lipid Nanoparticles | Dacarbazine | Chemotherapy | Enhancing drug retention in the skin, reducing side effects | Skin Cancer | [5] |
|  |  | Curcumin Derivative-Loaded Solid Lipid Nanoparticles | Curcumin Derivative | Chemotherapy | Enhancing drug stability and safety | Lung Cancer &Liver cancer | [6] |
|  |  | Multifunctional Biomimetic Nanoplatform | Ce6 and Ola | PDT & Chemotherapy &Immunochemotherapy | Co-delivered photosensitizing agents Ce6 and Olaparib to prevent DNA repair, activate the cGAS-STING signaling pathway to produce cytokines, induce ICD to elicit systemic anti-tumor immune responses | Breast Cancer | [7] |
|  | Polymer-Based Nanomaterials-Dendrimers | Dendrimer-Decorated Nanogels (PAMAM Dendrimer Decorated PVCL-GMA Nanogels) | DOX | Chemotherapy | Dendrimer modification renders the NGs with a positively charged surface and a neutral core, enhancing cellular internalization through cation-mediated active transport in acidic tumor regions | Cancer | [8] |
|  |  | Exfoliated Graphite-Based Electrochemical Immunosensor on a Dendrimer/Carbon Nanodot Platform | NA | Diagnosis | Detection of Carcinoembryonic Antigen by electrochemical method | Detection | [9] |
|  | Polymer-Based Nanomaterials-Polymeric Micelles | Redox-Sensitive Polymeric Micelles with Aggregation-Induced Emission | DOX | Chemotherapy | Redox sensitive polymer micelles trigger drug release via GSH-responsive disulfide bonds with aggregate induced emission properties for bioimaging | Breast Cancer | [10] |
|  |  | Dual-Triggered Peptide-Based Polymeric Micelles | DOX | Chemotherapy | Dual-responsive polymer micelles effectively target both extracellular and intracellular enzymes, enhancing the therapeutic efficacy of DOX | Breast Cancer | [11] |
|  |  | Polymeric Micelles with pH-Sensitive Fluorescent Marker | NA | Imaging | Rhodamine 6G conjugated with its FRET or PeT to NBD enables selective imaging and drug delivery of tumor cells through pH sensitivity in the range of 5.5-7.5 | A875 and K562 Cell | [12] |
|  |  | Thermo/pH Dual-Responsive Micelles Based on Host-Guest interaction | DOX | Chemotherapy | The host-guest interaction between benzimidazole-terminated graft copolymer and β-cyclodextrin-functionalized star block copolymer used to achieve both temperature and pH responsiveness | Breast Cancer MCF-7 Cell Line | [13] |
|  | Carbon-Based Nanomaterials-Carbon Nanotubes | Carbon Nanotube-Based Optoelectronic-Pulse Sensor Framework | NA | Diagnosis | Development of a photo-assisted alternating current pulse sensor based on CNTs pulsed under light irradiation to distinguish healthy cells from breast cancer cells | MCF-7 Cell Line | [14] |
|  |  | PEGylated Carbon Nanotubes Decorated with Silver Nanoparticles | NA | PTT | Silver nanoparticles decorated with PEGylated Carbon Nanotubes to induce PTT | Malignant Melanoma Tumors | [15] |
|  | Carbon-Based Nanomaterials-Graphene Oxide | Ternary Graphene Oxide/Chitosan/Silver Nanocomposites | NA | PTT | The surface of GO/Cs is decorated with AgNPs to enhance the efficacy of photothermal therapy PTT. | H460, HCT116, MDA-MB-468 and FaDu Cell Lines | [16] |
|  |  | Stimuli-Responsive Graphene Oxide and Methotrexate-loaded Magnetic Nanoparticles | MTX | PTT & Chemotherapy | GO-supported PEGylated superparamagnetic iron oxide (SPIONs) and MTX linked by stimulus-response linkers to target breast cancer cells and induce high cytotoxicity | Breast Cancer | [17] |
| **Inorganic Materials** | Gold Nanoparticles (AuNPs) | Gold Nanoparticles and Carboxymethyl Cellulose Nanocomposite | NA | Anticancer activity | Induce apoptosis and necrosis in liver cancer cells by increasing caspase-8 and -9 activity and decreasing VEGFR-2 | Breast Cancer | [18] |
|  |  | Ag_2_S/Ag Decorated ZnIn_2_S_4_/C_3_N_4_ 3D/2D Z-Scheme Heterostructures | NA | Diagnosis | The ultrasensitive detection of telomerase activity by using the Ag_2_S/Ag modified Z- scheme heterostructure and the photochemical signal amplified by Au/Cu^2+^- boron nitride nanozyme | HeLa, L02, MCF-7, HepG2, MEA Cell Line | [19] |
|  |  | Chemiluminescence-Derived Self-Powered Photoelectrochemical Immunoassay | NA | Ultrasensitive detection | Low abundance disease-associated proteins were detected by chemiluminescence derived self-powered photoimmunochemical assay | NA | [20] |
|  | Iron Oxide Nanoparticles | pH-responsive Fe_3_O_4_/chitosan/agarose double nanoemulsion | Curcumin | Chemotherapy | pH-responsive release of curcumin to avoid overdosing and reduce side effects, achieving tumor targeting through EPR effects and stability | Breast Cancer MCF-7 Cell Line | [21] |
|  |  | SPIONs | Crizotinib | Immunotherapy | Reshape lung TME into cytotoxic characteristics and recruit CD8+ T cells | NSCLC | [22] |
|  |  | Starch-coated iron oxide nanoparticles | NA | Immunotherapy | Magnetic Targeting, Immunotherapy Iron oxide nanoparticles enhance magnetic targeting to tumors, stimulate antitumor T-cell responses to improve immune-mediated tumor clearance. | Breast Cancer | [23] |
|  | Mesoporous Silica Nanoparticles | Thiolated Mesoporous Silica Nanoparticles as Immunoadjuvant | Mesoporous Silica Nanoparticles | Chemoimmunotherapy | Enhancing drug delivery and act as immunoadjuvants, boosting immune response alongside chemotherapy. | Bladder Cancer | [24] |
|  |  | Reactive Oxygen Species-Sensitive Biodegradable Mesoporous Silica Nanoparticles Harboring TheraVac | TheraVac | Chemoimmunotherapy | Mesoporous silica nanoparticles degrade in response to ROS, delivering TheraVac to induce tumor-specific immune response. | Colon Cancer | [25] |
|  |  | Hollow Mesoporous Silica Nanoparticles (HMSNs) | Cancer Vaccine | Immunotherapy | HMSNs with extra-large mesopores to enhance the loading and controlled release of cancer antigens, promoting an immune response and tumor immunotherapy. | Colon or Breast Cancer. | [26] |
|  | Quantum Dots | Carbon Quantum Dots-Based Nanozyme from Coffee | Carbon Quantum Dots, Ferroptosis Inducers | Immunotherapy | Nanozyme induces ferroptosis in cancer cells, activating antitumor immunity and enhancing therapeutic efficacy. | Liver Cancer | [27] |
|  |  | Chiral FA Conjugated CdTe/CdS Quantum Dots | FA, CdTe/CdS Quantum Dots | PTT | FA-conjugated quantum dots selectively target cancer cells, and upon light exposure, induce localized tumor ablation. | Breast and Ovarian Cancer | [28] |
|  |  | Bismuthene Functionalized with Red Phosphorus Quantum Dots | Bismuthene, Red Phosphorus Quantum dots | Theranostics (chemotherapy & imaging) | Bismuthene-based nanomaterials function as drug carriers and enhance therapeutic efficacy while enabling simultaneous imaging. | Cervical, Breast and Prostate Cancer | [29] |
| **Biological Materials** | Biomimetic Nanomaterials | Biomimetic Nanoerythrosome-Coated Aptamer–DNA Tetrahedron/Maytansine Conjugates | Maytansine | Chemotherapy | pH-responsive aptamer-DNA tetrahedron nanoconjugates to deliver Maytansine to HER2-positive cancer cells, inducing targeted cytotoxicity. | HER2-Positive Breast Cancer | [30] |
|  |  | Biomimetic Macrophage Membrane-Camouflaged Nanoparticles | Ferroptosis-Inducing Agents | Ferroptosis Therapy | Macrophage membrane-coated nanoparticles to induce ferroptosis in glioblastoma by promoting mitochondrial damage. | Glioblastoma | [31] |
|  |  | Senescent Cancer Cell Membrane-Based Biomimetic Nanovaccine | Cancer cell Membrane, Immune-Modulating Agents | Immunotherapy | Cancer cell membranes to enhance immune responses, boosting the effectiveness of cancer immunotherapy. | Melanoma | [32] |
|  |  | Engineered Biomimetic Nanoparticles | Various Metabolism-Targeting Agents | Metabolism-Based Synergistic Therapy | Engineered nanoparticles to target tumor metabolism pathways for synergistic therapy and enhanced drug delivery against glioblastoma. | Glioblastoma | [33] |
|  | Cell Membrane-Coated Nanoparticles | Cancer Cell-Mitochondria Hybrid Membrane-Coated Nanomedicines | Gboxin | Targeted Chemotherapy | Gboxin-loaded nanoparticles, coated with hybrid cancer cell-mitochondria membranes to enhance tumor targeting and induce mitochondrial dysfunction. | Glioblastoma | [34] |
|  |  | Genetically Engineered Membrane-Coated Nanoparticles | Chemotherapeutics | Enhanced Targeted Drug Delivery | Genetic engineering of cell membranes to improve targeting, internalization, and therapeutic efficacy of nanoparticles. | Ovarian Cancer | [35] |
|  |  | Neutrophil Membrane-Coated Gold Nanoparticles | STING Agonist | Immunotherapy & Radiotherapy | Neutrophil membrane-coated gold nanoparticles to deliver STING agonists, activating immune response and enhancing radiotherapy-induced tumor inhibition. | Breast Cancer | [36] |
|  | Exosomes | Engineered Exosomes | lncRNA MEG3 | Gene Therapy | Delivering lncRNA MEG3 to osteosarcoma cells, restoring tumor-suppressor functions and inhibiting tumor growth. | Osteosarcoma | [37] |
|  |  | HEK-293T Engineered Exosomes | DOX, ^131^I | Chemotherapy & Internal Radiation | Delivering chemotherapeutics and irradiation agents to tumors, enabling synergistic tumor-targeted treatment. | Thyroid Cancer | [38] |
|  |  | Engineered Exosome Vaccine | Vaccine | Immunotherapy (Vaccine) | Functions as an *in situ* dendritic cell (DC)-primed vaccine, boosting immune responses against tumor cells. | Breast Cancer | [39] |
|  |  | Exosomes with exoASO-STAT6 | Genetic Reprogramming Agent | Gene Therapy & Immunotherapy | Reprograming TAMs to suppress tumor growth and enhance antitumor immunity. | Colorectal Cancer  Liver Cancer | [40] |
|  | DNA nanomaterials | 84bp-TDNISD/56MESS | Interferon Stimulatory DNA + Toxins | Chemoimmunotherapy | Delivering potent toxins to cancer cells and activates the cGAS-STING pathway to stimulate an antitumor immune response. | Breast Cancer | [41] |
|  |  | Autocatalytic Multicomponent DNAzyme Nanomachine | DNAzyme Components | PTT | Tumor-specific DNAzyme activation amplifying therapeutic effects; enhances photothermal sensitivity by disrupting cellular processes. | Pancreatic Cancer | [42] |
|  |  | DNA nanocluster | DNA Components | Radiotherapy & Immunotherapy | Enhances radiotherapy by amplifying DNA damage, promoting immunogenic cell death, and activating antitumor immune responses. | Colon and Breast Cancer | [43] |
|  |  | Protein-Conjugated DNA Nanoplatform | Proteins + DNA Therapeutics | Synergistic Chemotherapy | Combines targeted protein delivery with DNA-based therapies to enhance tumor-specific cytotoxicity and disrupt cellular pathways. | Breast, Lung, and Liver cancer | [44] |
| **Other/Hybrid Materials**  **Other/Hybrid Materials** | Metal-Organic Frameworks (MOFs) | Cu(Ⅱ)-MOF nanocomposite | Nanozymes, Anti-PD-L1 antibody | Cuproptosis Induction | Oxidative damage  Glutathione peroxidase-like activity,  induction of cuproptosis ICD, and  tumor microenvironment modulation | Breast Cancer | [45] |
|  |  | Titanium-Based Nanomaterials | Radiosensitizer | Radio-enhancement | Catalytic ROS generation with  broad-spectrum radiocatalytic activity | Soft Tissue Sarcoma | [46] |
|  |  | Diselenide MOF-Based Nanomotor | Carbon Monoxide | PTT | Carbon monoxide release induces localized cytotoxicity; NIR-II light activates PTT for tumor ablation. | Breast Cancer | [47] |
|  |  | Pitaya-Inspired MOF Nanozyme | Cu (Ⅱ) | Nanocatalytic Therapy, PTT & imaging | Induces cuproptosis (copper-mediated cell death), enhances ROS generation for nanocatalytic therapy, and utilizes NIR light for photothermal ablation | Lung Cancer | [48] |
|  | Core-Shell Nanoparticles | Core-Shell Nanoscale Coordination Polymer Nanoparticles | Sialyltransferase Inhibitor | Cancer Metastasis Inhibition | Self-assembled nanoparticles delivering a sialyltransferase inhibitor, blocking sialylation processes that promote metastasis and tumor cell adhesion. | Breast Cancer, Lung Cancer | [49] |
|  |  | Layer-by-Layer Core-Shell Nanoparticles | Histone Deacetylase Inhibitors | Central Nervous System -Targeted Chemotherapy | Enhances CNS drug delivery by crossing the blood-brain barrier; histone deacetylase inhibitors modulate gene expression to inhibit tumor growth and induce apoptosis. | Diffuse Midline Gliomas | [50] |
|  |  | Dual-Responsive Core–Shell Tecto Dendrimers | CRISPR/Cas9 Components | Gene Editing & Immunotherapy | Efficiently delivers CRISPR/Cas9 for targeted gene editing in cancer cells; enhances immune checkpoint blockade therapy by modulating immune response. | Melanoma | [51] |
|  |  | Mesoporous Magnetic Fe_3_O_4_/BioMOF-13 Nanostructure | DOX | Targeted Chemotherapy | Core/shell nanostructure enabling magnetic targeting and controlled release of DOX; enhances tumor-specific cytotoxicity while reducing off-target effects. | Breast Cancer | [52] |

**References**

[1] K. Luo, L. Yang, C. Yan, et al., A dual-targeting liposome enhances triple-negative breast cancer chemoimmunotherapy through inducing immunogenic cell death and inhibiting STAT3 activation. Small. 19 (2023) 2302834.

[2] T. Zhu, Z. Chen, G. Jiang, et al., Sequential targeting hybrid nanovesicles composed of chimeric antigen receptor T-Cell-Derived exosomes and liposomes for enhanced cancer immunochemotherapy. ACS Nano. 17 (2023) 16770-16786.

[3] W. Lu, W. Liu, A. Hu, et al., Combinatorial polydopamine-liposome nanoformulation as an effective anti-breast cancer therapy. Int. J. Nanomedicine. 18 (2023) 861-879.

[4] M. Qiu, J. Wang, J. Bai, et al., Dual-ligand-functionalized liposomes based on glycyrrhetinic acid and crgd for hepatocellular carcinoma targeting and therapy. Mol. Pharm. 20 (2023) 1951-1963.

[5] S. Bhattacharya, and S. Sharma, Dacarbazine-encapsulated solid lipid nanoparticles for skin cancer: physical characterization, stability, in-vivo activity, histopathology, and immunohistochemistry. Front. Oncol. 13 (2023) 1102269.

[6] K. Li, C. Pi, J. Wen, et al., Formulation of the novel structure curcumin derivative–loaded solid lipid nanoparticles: synthesis, optimization, characterization, and anti-tumor activity screening in vitro. Drug Deliv. 29 (2022) 2044-2057.

[7] H. Wu, X. Du, J. Xu, et al., Multifunctional biomimetic nanoplatform based on photodynamic therapy and DNA repair intervention for synergistic treatment of breast cancer. SSRN Electron. J. (2022) 551-565.

[8] X. Li, Z. Ouyang, H. Li, et al., Dendrimer-Decorated Nanogels: efficient nanocarriers for biodistribution in vivo and chemotherapy of ovarian carcinoma. Bioact. Mater. 6 (2021) 3244-3253.

[9] A.O. Idris, N. Mabuba, and O.A. Arotiba, An exfoliated graphite-based electrochemical immunosensor on a dendrimer/carbon nanodot platform for the detection of carcinoembryonic antigen cancer biomarker. Biosens. 9 (2019) 39.

[10] C. Sun, J. Lu, J. Wang, et al., Redox-sensitive polymeric micelles with aggregation-induced emission for bioimaging and delivery of anticancer drugs. J. Nanobiotechnol. 19 (2021) 1-15.

[11] Y. Liu, Z. Ren, X. Zhang, et al., Dual-triggered peptide-based polymeric micelles enhance doxorubicin delivery for targeted cancer therapy. ACS Appl. Nano Mater. 7 (2024) 14380-14391.

[12] I.D. Zlotnikov, A.A. Ezhov, and E.V. Kudryashova, pH-sensitive fluorescent marker based on Rhodamine 6G conjugate with its FRET/PeT pair in “Smart” polymeric micelles for selective imaging of cancer cells. Pharmaceutics. 16 (2024) 1007.

[13] F. Adeli, F. Abbasi, M. Babazadeh, et al., Thermo/pH dual-responsive micelles based on the host–guest interaction between benzimidazole-terminated graft copolymer and β-cyclodextrin-functionalized star block copolymer for smart drug delivery. J. Nanobiotechnol. 20 (2022) 91.

[14] S.S.Y. Chan, D. Lee, M.P. Meivita, et al., Ultrasensitive detection of MCF-7 cells with a carbon nanotube-based optoelectronic-pulse sensor framework. ACS Omega. 7 (2022) 18459-18470.

[15] M.A. Behnam, F. Emami, and Z. Sobhani, PEGylated carbon nanotubes decorated with silver nanoparticles: fabrication, cell cytotoxicity, and application in photo thermal therapy. Iran. J. Pharm. Res. 20 (2021) 91-104.

[16] M.A. Ramadan, M. Sharaky, S. Gad, et al., Anticancer effect and laser photostability of ternary graphene oxide/chitosan/silver nanocomposites on various cancer cell lines. Nanomed. 19 (2024) 709-722.

[17] M. Dolatkhah, N. Hashemzadeh, J. Barar, et al., Stimuli-responsive graphene oxide and methotrexate-loaded magnetic nanoparticles for breast cancer-targeted therapy. Nanomed. 16 (2021) 2155-2174.

[18] A.S. Doghish, A.H. Hashem, A.M. Shehabeldine, et al., Nanocomposite based on gold nanoparticles and carboxymethyl cellulose: synthesis, characterization, antimicrobial, and anticancer activities. J. Drug Deliv. Sci. Technol. 77 (2022) 103874.

[19] J.-H. Zhu, H. Gou, T. Zhao, et al., Ultrasensitive photoelectrochemical aptasensor for detecting telomerase activity based on Ag_2_S/Ag Decorated ZnIn_2_S_4_/C_3_N_4_ 3D/2D Z-scheme heterostructures and amplified by Au/Cu^2+^-boron-nitride nanozyme. Biosens. Bioelectron. 203 (2022) 114048.

[20] Z. Yu, H. Gong, Y. Li, et al., Chemiluminescence-derived self-powered photoelectrochemical immunoassay for detecting a low-abundance disease-related protein. Anal. Chem. 93 (2021) 13389-13397.

[21] M. Pourmadadi, M. Ahmadi, and F. Yazdian, Synthesis of a novel pH-responsive Fe_3_O_4_/chitosan/agarose double nanoemulsion as a promising Nanocarrier with sustained release of curcumin to treat MCF-7 cell line. Int. J. Biol. Macromol. 235 (2023) 123786.

[22] N.K. Horvat, S. Chocarro, O. Marques, et al., Superparamagnetic iron oxide nanoparticles reprogram the tumor microenvironment and reduce lung cancer regrowth after crizotinib treatment. ACS Nano. 18 (2024) 11025-11041.

[23] P. Korangath, L. Jin, C.T. Yang, et al., Iron oxide nanoparticles inhibit tumor progression and suppress lung metastases in mouse models of breast cancer. ACS Nano. 18 (2024) 10509-10526..

[24] C.C. Chen, Y.C. Fa, Y.Y. Kuo, et al., Thiolated mesoporous silica nanoparticles as an immunoadjuvant to enhance efficacy of intravesical chemotherapy for bladder cancer. Adv. Sci. 10 (2023) 2204643.

[25] Y. Huang, S. Nahar, M.D.M. Alam, et al., Reactive oxygen species-sensitive biodegradable mesoporous silica nanoparticles harboring TheraVac elicit tumor-specific immunity for colon tumor treatment. ACS Nano. 17 (2023) 19740-19752.

[26] J.Y. Lee, M.K. Kim, T.L. Nguyen, et al., Hollow mesoporous silica nanoparticles with extra-large mesopores for enhanced cancer vaccine. ACS Appl. Mater. Interfaces. 12 (2020) 34658-34666.

[27] L. Yao, M.-M. Zhao, Q.-W. Luo, et al., Carbon quantum dots-based nanozyme from coffee induces cancer cell ferroptosis to activate antitumor immunity. ACS Nano. 16 (2022) 9228-9239.

[28] G. Li, X. Zhang, X. Fei, et al., Chiral FA conjugated CdTe/CdS quantum dots for selective cancer ablation. ACS Nano. 16 (2022) 12991-13001.

[29] H. Song, J. Wang, B. Xiong, et al., Biologically Safe, Versatile, and smart bismuthene functionalized with a drug delivery system based on red phosphorus quantum dots for cancer theranostics. Angew. Chem. Int. Ed. 61 (2022) e202117679.

[30] W. Ma, Y. Yang, J. Zhu, et al., Biomimetic nanoerythrosome‐coated aptamer–DNA tetrahedron/maytansine conjugates: pH‐responsive and targeted cytotoxicity for HER2‐positive breast cancer. Adv. Mater. 34 (2022) 2109609.

[31] Z. Cao, X. Liu, W. Zhang, et al., Biomimetic macrophage membrane-camouflaged nanoparticles induce ferroptosis by promoting mitochondrial damage in glioblastoma. ACS Nano. 17 (2023) 23746-23760.

[32] C. Yang, Y. Chen, J. Liu, et al., Leveraging senescent cancer cell membrane to potentiate cancer immunotherapy through biomimetic nanovaccine. Adv. Sci. 11 (2024) 2400630.

[33] G. Lu, X. Wang, F. Li, et al., Engineered biomimetic nanoparticles achieve targeted delivery and efficient metabolism-based synergistic therapy against glioblastoma. Nat. Commun. 13 (2022) 4214.

[34] Y. Zou, Y. Sun, Y. Wang, et al., Cancer cell-mitochondria hybrid membrane coated Gboxin loaded nanomedicines for glioblastoma treatment. Nat. Commun. 14 (2023) 4577.

[35] N. Krishnan, Y. Jiang, J. Zhou, et al., A modular approach to enhancing cell membrane-coated nanoparticle functionality using genetic engineering. Nat. Nanotechnol. 19 (2023) 345-353.

[36] D. Lu, W. Li, J. Tan, et al., STING Agonist Delivered by neutrophil membrane-Coated gold nanoparticles exerts synergistic tumor inhibition with radiotherapy. ACS Appl. Mater. Interfaces. 16 (2024) 53474-53488.

[37] X. Huang, W. Wu, D. Jing, et al., Engineered exosome as targeted lncRNA MEG3 delivery vehicles for osteosarcoma therapy. J. Control. Release. 343 (2022) 107-117.

[38] C. Wang, N. Li, Y. Li, et al., Engineering a HEK-293T exosome-based delivery platform for efficient tumor-targeting chemotherapy/internal irradiation combination therapy. J. Nanobiotechnol. 20 (2022) 247.

[39] L. Huang, Y. Rong, X. Tang, et al., Engineered exosomes as an in situ DC-primed vaccine to boost antitumor immunity in breast cancer. Mol. Cancer. 21 (2022) 45.

[40] S. Kamerkar, C. Leng, O. Burenkova, et al., Exosome-mediated genetic reprogramming of tumor-associated macrophages by exoASO-STAT6 leads to potent monotherapy antitumor activity. Sci. Adv. 8 (2022) eabj7002.

[41] L. Zhang, Y. Wang, J. Karges, et al., Tetrahedral DNA nanostructure with interferon stimulatory DNA delivers highly potent toxins and activates the cGAS‐STING pathway for robust chemotherapy and immunotherapy. Adv. Mater. 35 (2022) 2210267.

[42] J. Yan, X. Ma, D. Liang, et al., An autocatalytic multicomponent DNAzyme nanomachine for tumor-specific photothermal therapy sensitization in pancreatic cancer. Nat. Commun. 14 (2023) 6905.

[43] Y. Xie, H. Li, L. Xu, et al., DNA nanoclusters combined with One‐Shot radiotherapy augment cancer immunotherapy efficiency. Adv. Mater. 35 (2023) 2208546.

[44] D. Liu, Y. Chen, Q. Wang, et al., Tailored protein-conjugated DNA nanoplatform for synergistic cancer therapy. J. Control. Release. 346 (2022) 250-259.

[45] X. Lu, W. Deng, S. Wang, et al., PEGylated Elesclomol@Cu(Ⅱ)-based Metal‒organic framework with effective nanozyme performance and cuproptosis induction efficacy for enhanced PD-L1-based immunotherapy. Mater. Today Bio. 29 (2024) 101317.

[46] L.R.H. Gerken, C. Beckers, B.A. Brugger, et al., Catalytically active Ti‐based nanomaterials for hydroxyl radical mediated clinical X‐ray enhancement. Adv. Sci. (2024) 2406198.

[47] R.Z. Tian, Z.R. Zhang, L.P. Song, et al., A diselenide MOF-based nanomotor dual-driven by carbon monoxide and near-infrared-II light for multimodal tumor-targeted therapy. Sci. China Chem. (2024) 1-18.

[48] Q. Yue, Q. Zeng, Q. Guo, et al., Pitaya‐inspired metal‐organic framework nanozyme for multimodal imaging‐guided synergistic cuproptosis, nanocatalytic therapy, and photothermal therapy. Adv. Healthcare Mater. (2024) 2402915.

[49] X. Zhang, C.-H. Xu, J. Mo, et al., Self-assembled core–shell nanoscale coordination polymer nanoparticles carrying a sialyltransferase inhibitor for cancer metastasis inhibition. ACS Appl. Mater. Interfaces. 15 (2023) 7713-7724.

[50] M.H. Marand, E. Han, S.H. Kim, et al., Surg-03. Layer-by-layer core-shell nanoparticles for the delivery of HDAC inhibitors to the central nervous system. Neuro-Oncol. 25 (2023) i72-i72.

[51] J. Liu, G. Li, H. Guo, et al., Dual-responsive core–shell tecto dendrimers enable efficient gene editing of cancer cells to boost immune checkpoint blockade therapy. ACS Appl. Mater. Interfaces. 15 (2023) 12809-12821.

[52] R. Taheri-Ledari, S. Zarei-Shokat, F.S. Qazi, et al., A mesoporous magnetic Fe_3_O_4_/BioMOF-13 with a core/shell nanostructure for targeted delivery of doxorubicin to breast cancer cells. ACS Appl. Mater. Interfaces (2023).
